# *Complement C7* is a novel risk gene for Alzheimer's disease in Han Chinese

**DOI:** 10.1093/nsr/nwy127

**Published:** 2018-11-05

**Authors:** Deng-Feng Zhang, Yu Fan, Min Xu, Guihong Wang, Dong Wang, Jin Li, Li-Li Kong, Hejiang Zhou, Rongcan Luo, Rui Bi, Yong Wu, Guo-Dong Li, Alzheimer's Disease Neuroimaging Initiative (ADNI), Ming Li, Xiong-Jian Luo, Hong-Yan Jiang, Liwen Tan, Chunjiu Zhong, Yiru Fang, Chen Zhang, Nengyin Sheng, Tianzi Jiang, Yong-Gang Yao

**Affiliations:** 1 Key Laboratory of Animal Models and Human Disease Mechanisms of the Chinese Academy of Sciences & Yunnan Province, Kunming Institute of Zoology, Chinese Academy of Sciences, Kunming 650223, China; 2 Center for Excellence in Animal Evolution and Genetics, Chinese Academy of Sciences, Kunming 650223, China; 3 Kunming College of Life Science, University of Chinese Academy of Sciences, Kunming 650204, China; 4 Center for Neurodegenerative Diseases, Department of Neurology, Beijing Tiantan Hospital, Capital Medical University, Beijing 100050, China; 5 Brainnetome Center, Institute of Automation, Chinese Academy of Sciences, Beijing 100190, China; 6 National Laboratory of Pattern Recognition, Institute of Automation, Chinese Academy of Sciences, Beijing 100190, China; 7 CAS Center for Excellence in Brain Science and Intelligence Technology, Chinese Academy of Sciences, Shanghai 200031, China; 8 Department of Psychiatry, the First Affiliated Hospital of Kunming Medical University, Kunming 650032, China; 9 Mental Health Institute of the Second Xiangya Hospital, Central South University, Changsha 410011, China; 10 Department of Neurology, Zhongshan Hospital, Fudan University, Shanghai 200032, China; 11 Division of Mood Disorders, Shanghai Mental Health Center, Shanghai Jiao Tong University School of Medicine, Shanghai 200030, China; 12 State Key Laboratory of Genetic Resources and Evolution, Kunming Institute of Zoology, Chinese Academy of Sciences, Kunming 650223, China; 13 KIZ–CUHK Joint Laboratory of Bioresources and Molecular Research in Common Diseases, Kunming Institute of Zoology, Chinese Academy of Sciences, Kunming 650223, China

**Keywords:** Alzheimer's disease, whole-exome sequencing, C7, neuroimaging, complement system

## Abstract

Alzheimer's disease is the most common neurodegenerative disease, and has a high level of genetic heritability and population heterogeneity. In this study, we performed the whole-exome sequencing of Han Chinese patients with familial and/or early-onset Alzheimer's disease, followed by independent validation, imaging analysis and function characterization. We identified an exome-wide significant rare missense variant rs3792646 (p.K420Q) in the *C7* gene in the discovery stage (*P* = 1.09 × 10^−6^, odds ratio = 7.853) and confirmed the association in different cohorts and a combined sample (1615 cases and 2832 controls, *P_combined_* = 2.99 × 10^−7^, odds ratio = 1.930). The risk allele was associated with decreased hippocampal volume and poorer working memory performance in early adulthood, thus resulting in an earlier age of disease onset. Overexpression of the mutant p.K420Q disturbed cell viability, immune activation and β-amyloid processing. Electrophysiological analyses showed that the mutant p.K420Q impairs the inhibitory effect of wild type C7 on the excitatory synaptic transmission in pyramidal neurons. These findings suggested that *C7* is a novel risk gene for Alzheimer's disease in Han Chinese.

## INTRODUCTION

Alzheimer's disease is the most common neurodegenerative disease in the elderly and is becoming a serious global health problem [1–3]. It is characterized by cognitive impairment resulting from extracellular β-amyloid (Aβ) plaques, intracellular neurofibrillary tangles (hyperphosphorylated tau) and cerebral atrophy [1–3]. Both genetic and environmental factors contribute to the onset and development of the disease, and its heritability is reported to be up to 0.79 [4–6]. Previous linkage analyses have revealed genes involved in the production of Aβ plaques, namely *APP* (Aβ precursor protein), *PSEN1* (Presenilin-1) and *PSEN2* (Presenilin-1), as the causal genes for early-onset familial Alzheimer's disease [5,7–15]. However, mutations of these genes are mainly associated with the autosomal dominant types and account for less than 5% of the total number of cases [5,16]. In fact, it is believed that in most cases, the disease is polygenic and there are other causal and/or susceptibility genes remaining to be discovered [4,17]. Recent genome-wide association studies (GWASs) have reported two dozen Alzheimer's susceptibility genes in populations of European ancestry, including *APOE*, *BIN1*, *CLU* and *RIN3* [18,19]. Nevertheless, most of the GWAS loci are non-coding common variants/SNPs (single nucleotide polymorphisms) with unknown function and show small to moderate effect sizes (odds ratio [OR] < 1.2). Since these GWAS hits can only explain about 16% of the total phenotypic variance [17], the missing heritability remains to be explained by other underlying variants (especially functionally causative variants) [20].

Recent advances in next-generation sequencing technologies offer powerful tools for the discovery of rare causal variants with larger effect sizes in Alzheimer's disease [21], and previous studies have identified *UNC5C* [22], *TREM2* [23,24], *PLD3* [25,26], *PLCG2* and *ABI3* [27] to be the top candidate genes harboring such variants. However, despite the successes in applying next-generation sequencing technologies, population heterogeneity has limited the success in characterizing the genetic basis of Alzheimer's disease [28,29]. For example, many of top hits in the European populations identified by GWASs or next-generation sequencing technologies cannot be validated in Chinese populations [26,30–32]. The investigation of the genetic susceptibility of Alzheimer's disease at the whole-genome level in Han Chinese, the largest ethnic population in the world, which has the greatest number of Alzheimer's disease sufferers [33,34], is therefore urgently needed. To this end, we have performed whole-exome sequencing (WES) of Han Chinese patients with Alzheimer's disease to identify novel susceptibility genes.

## RESULTS

### Identification of *C7* as a novel Alzheimer's risk gene in Han Chinese

**Figure 1.**
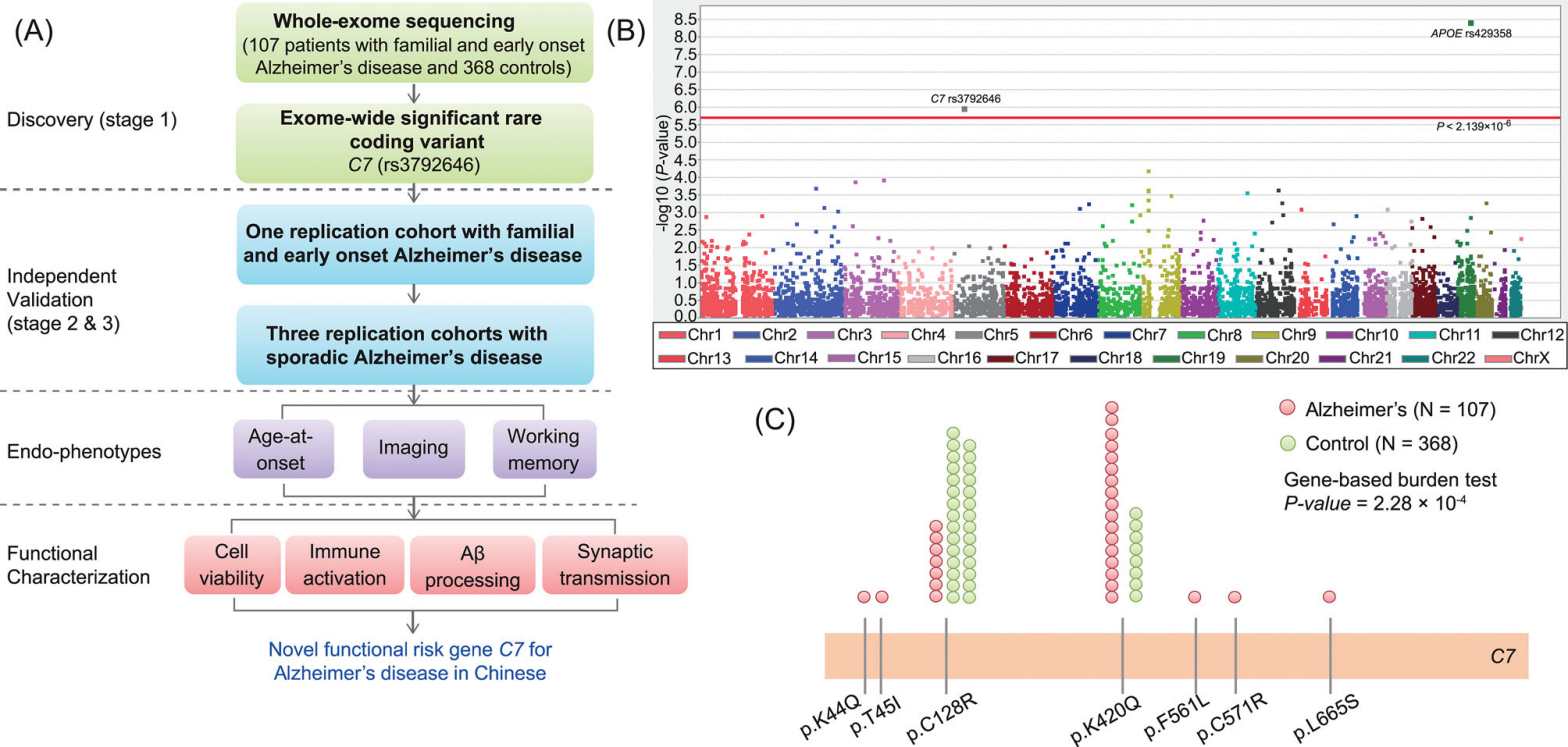
Identification of *C7* variant rs3792646 (p.K420Q) in Han Chinese patients with early-onset and/or familial Alzheimer's disease. (A) Workflow of the current study. (B) Manhattan plot of the exome-wide single site association in 107 cases and 368 population controls for rare and low-frequency (MAF < 5%) coding (missense, nonsense and splice site) variants, with *APOE* rs429358 (which defines the ε4 allele) being a positive control. Red line, exome-wide significance. (C) Rare damaging variants of *C7* in Chinese Alzheimer's cases and controls. *P*-value, gene-based burden test.

We used an extreme phenotype sampling strategy for WES to increase the likelihood of identifying true disease-related variants [35,36], followed by independent validations and functional characterization (Fig. 1A). In total, 107 unrelated patients with an early age at onset (AAO) of Alzheimer's disease (AAO ≤ 55) and/or a positive familial history were selected from over 1000 genetically unrelated patients from East and Southwest China [26,32,37–41]. In addition, 160 in-house non-dementia individuals [42], together with the whole-genome data of Han Chinese in Beijing (n = 103) and Southern Han Chinese (n = 105) from the 1000 Genomes Project phase 3 [43], were combined as the initial population control (n = 368), based on the fact that principal component (PC) analysis showed no apparent population stratification between the studied subjects and the reference Chinese populations from phase 3 of the 1000 Genomes Project [43] (Supplementary Fig. S1). Nonsense, frameshift, splice site and missense variants, which were predicted to be damaging by at least one of the five algorithms (PolyPhen2 HumDiv and HumVar [44], LRT [45], MutationTaster [46] and SIFT [47,48]), were defined as functional. As we aimed to identify novel rare coding variants that were associated with Alzheimer's disease or enriched in patients, we filtered out the common variants and obtained 23 373 rare or low-frequency coding variants with a minor allele frequency (MAF) < 5% in the 368 pooled population controls [42,43]. The Bonferroni correction-based threshold for the exome-wide significance was thus set as *P* < 2.139 × 10^−6^ (0.05/23373).

We were able to successfully validate the previously reported association of *APOE* ε4 with Alzheimer's disease [18] (rs429358, *P* = 3.41 × 10^−9^, OR = 3.59) in our initial WES screening stage (Fig. 1B and Supplementary Table S1), suggesting the reliability of the current extreme phenotype sampling approach. One rare variant in the complement *C7* gene, rs3792646, leading to the missense mutant p.K420Q with a predicted damaging effect (Combined Annotation-Dependent Depletion score = 20.3) [49], was the only exome-wide significant hit showing an association with susceptibility to Alzheimer's disease (*P* = 1.09 × 10^−6^, OR = 7.853, Fisher's exact test; Fig. 1B, Table 1 and Supplementary Table S1), with the exception of *APOE* rs429358. When the 107 patients (MAF = 0.08) were compared with 4327 East Asians from the Exome Aggregation Consortium (ExAC) [50] (MAF = 0.036; *P* = 2.95 × 10^−3^, OR = 2.292, Fisher's exact test) or 11 670 Chinese individuals from the CONVERGE (China, Oxford and Virginia Commonwealth University Experimental Research on Genetic Epidemiology) consortium [51] (MAF = 0.029; *P* = 2.200 × 10^−4^, OR = 2.884, Fisher's exact test), the positive association between rs3792646 and Alzheimer's disease survived (Table 1). To avoid a false-positive result, we performed logistic regression analyses with adjustment of the first three PCs (PC1, PC2 and PC3) (Supplementary Fig. S1), APOE ε4 status and sex as the covariate(s), separately or together. The association of *APOE* ε4 with Alzheimer's disease remained significant at the exome-wide level after adjustment with PC1–3 (*P_adj__-PC_* = 1.95 × 10^−7^, OR*_adj__-PC_* = 3.20). The *C7* variant rs3792646 remained one of the top hits after adjustment using different covariates (*P_adj__-PC_* = 9.508 × 10^−5^, *P_adj__-sex_* = 7.30 × 10^−6^, *P_adj__-APOE_* = 5.0 × 10^−4^ and *P_adj__-PC-sex-APOE_* = 9.90 × 10^−4^), although no exome-wide significant hits were observed, partly because of the small sample size (Supplementary Table S1). We list the top 100 rare functional variants showing suggestive associations with Alzheimer's disease (Fisher's exact test, *P* < 0.01) in Supplementary Table S1. The summary statistics can be freely accessed through the webserver AlzData (http://www.alzdata.org/exome.html) [52].

**Table 1.** Identification and validation of the association between *C7* variant rs3792646 and Alzheimer's disease in Han Chinese.

			Alzheimer		Control				
Stage	Type	Region	Sample size	C/A allele	Sample size	C/A allele	*P*-value	OR	95% CI
1	Early-onset/familial	WES^a^	107	17/197	368	8/728	**1.09 × 10^−^^6^**	7.853	3.340–18.464
		ExAC	-	-	4327	295/7836	2.95 × 10^−3^	2.292	1.378–3.813
		CONVERGE	-	-	11 670	677/22 623	2.20 × 10^−4^	2.884	1.747–4.761
2	Early-onset/familial	North (Beijing)	103	11/195	368	8/728	6.10 × 10^−4^	5.133	2.037–12.937
Combined 1 and 2	Early-onset/familial		210	28/392	368	8/728	**3.73 × 10^−^^7^**	6.500	2.934–14.398
3	Sporadic	East	587	44/1130	274	7/541	3.73 × 10^−3^	3.009	1.347–6.725
	Sporadic	Southwest	583	45/1121	2190	108/4272	1.19 × 10^−2^	1.588	1.115–2.262
	Sporadic	Southcentral	235	16/454	2190	108/4272	0.218	1.394	0.817–2.378
	Sporadic	Pooled South^b^	818	61/1575	2190	108/4272	1.08 × 10^−2^	1.532	1.113–2.108
	Sporadic early-onset^c^		248	21/475	2464	115/4813	1.51 × 10^−2^	1.800	1.151–2.974
Combined 1–3	All early-onset		421	37/805	2832	123/5541	3.10 × 10^−4^	2.066	1.419–3.008
	All late-onset		1194	96/2292	2832	123/5541	8.11 × 10^−6^	1.883	1.434–2.472
	All cases		1615	133/3097	2832	123/5541	**2.99 × 10^−^^7^**	1.930	1.503–2.479
European	Sporadic	ADNI	296	1/591	281	0/562	NA	NA	NA
	Sporadic	ADSP	5815	0/11 630	4755	1/9509	NA	NA	NA

Note: The same control sample (n = 368) was used in stage 1 and stage 2. In stage 3, the same control sample (n = 2190) was used for comparison with cases from Southwest China and Southcentral China, respectively. The ADNI European sample was taken from the ADNI dataset [54]; the ADSP European sample was taken from the ADSP through the dbGaP (phs000572.v7.p4). Data of 4327 East Asians from the ExAC [50] and data of 11 670 Chinese individuals in the CONVERGE Consortium [51] were retrieved as the reference controls. C/A allele, risk allele/reference allele; *P*-value, Fisher’s exact test; OR, odds ratio of effect (minor) allele; CI, confidence interval; NA, not applicable. A total of 23 373 functional variants with low allele frequency (MAF < 5%) were used in the analysis, with a threshold for the exome-wide significance of *P* < 2.139 × 10^−6^ (Bonferroni corrected: 0.05/23 373). The exome-wide significant *P*-values are marked in bold.

^a^Logistic regression analysis was also performed for stage 1 samples. Suggestive associations of rs3792646 with Alzheimer's disease were observed after adjustment with different covariates: PC1-, PC2- and PC3- adjusted *P* = 9.51 × 10^−5^, OR = 5.731; *APOE* ε*4*-adjusted *P* = 5.36 × 10^−4^, OR = 5.107; sex-adjusted *P* = 7.29 × 10^−6^, OR = 8.716; PCs, *APOE* ε4 and sex-adjusted *P* = 9.90 × 10^−4^, OR = 5.382.

^b^Pooled South - Sporadic patients from Southwest and Southcentral China.

^c^Patients with early-onset Alzheimer's disease from East, Southwest and Southcentral China.

In addition to the single-site evidence, the gene-level association based on the burden test showed that *C7* had an enrichment of rare missense variants in Alzheimer's patients compared with controls (*P* = 2.28 × 10^−4^; Fig. 1C). The SNP-set (Sequence) Kernel Association Tests (SKAT) [53] yielded an even stronger association (*P* = 5.83 × 10^−7^) of the combined effects of rare *C7* variants. When rs3792646 was excluded from the burden test, the significance of enrichment disappeared, suggesting that the signal might be driven by rs3792646. As there was a variant, rs2271708 (p.C128R), overrepresenting in controls (Fig. 1C), we recalculated the burden test excluding both rs3792646 and rs2271708, and observed a marginally significant enrichment of rare variants in cases (*P* = 0.02), suggesting the existence of multiple effect alleles in *C7*.

### Validation of the association of rs3792646 with Alzheimer's disease in Han Chinese

To validate the association between *C7* rs3792646 and early-onset and familial Alzheimer's disease identified during the discovery WES screen (stage 1), we sequenced this SNP in an independent Han Chinese sample with early-onset and/or familial Alzheimer's disease from Beijing (stage 2, n = 103 cases). The association of rs3792646 with Alzheimer's disease could be well validated (*P* = 6.10 × 10^−4^, OR = 5.133, Table 1). Combing these two samples of patients with early-onset and/or familial Alzheimer's disease together, we observed a stronger association of rs3792646 with Alzheimer's disease (*P* = 3.73 × 10^−7^, OR = 6.500).

We then attempted to validate the association between rs3792646 and Alzheimer's disease in Chinese cohorts of sporadic patients (stage 3): the East China cohort contains 587 sporadic cases and 274 geographically matched controls, and the Southwest China cohort contains 583 sporadic cases and 2190 geographically matched controls. We also analyzed a patient sample from Hunan, Southcentral China (n = 235 sporadic cases). Positive associations were observed in the East China cohort (*P* = 3.73 × 10^−3^, OR = 3.009, Fisher's exact test) and the Southwest China cohort (*P* = 1.19 × 10^−2^, OR = 1.588, Fisher's exact test). In the sample from Hunan Province, Southcentral China, no association with Alzheimer's disease was observed (*P* = 0.218, OR = 1.394, Fisher's exact test), but the risk effect remained in this relatively small sample. When we combined the subjects from Southwest and Southcentral China together (Pooled South, Table 1), a positive association was observed (*P* = 1.08 × 10^−2^, OR = 1.532, Fisher's exact test). Though the associations from single validation cohorts did not reach exome-wide significance, combining all samples from stage 1 to stage 3 together resulted in an exome-wide significant association between rs3792646 and Alzheimer's risk with a considerably large effect size (*P_combined_* = 2.99 × 10^−7^, OR = 1.930).

Notably, we observed positive associations in both early-onset (AAO ≤ 65 years old; *P* = 3.10 × 10^−4^, OR = 2.066) and late-onset subjects (AAO > 65 years old; *P* = 8.11 × 10^−6^, OR = 1.883), with a stronger effect size in the early-onset patients (Table 1). When the patients were divided into different groups according to their *APOE* ε4 status, we observed positive associations of rs3792646 with Alzheimer's risk in both *APOE* ε4 carriers and non-carriers (Supplementary Table S2), and a stronger association was found in the *APOE* ε4 carriers (*P_combined_* = 1.43 × 10^−5^, OR = 3.651) than non-carriers (*P_combined_* = 1.22 × 10^−3^, OR = 1.770) (Supplementary Table S2).

### Association of rs3792646 with Alzheimer's disease might be Chinese-specific

While we have confirmed the association between *C7* rs3792646 with Alzheimer's disease in Han Chinese, it is unclear whether it is Chinese-specific or not. We therefore re-analyzed the whole-genome sequencing data of 812 individuals of European ancestry from the Alzheimer's Disease Neuroimaging Initiative (ADNI) dataset [54]. There were six *C7* mutation carriers (including three rare damaging missense variants; Supplementary Table S3) in 296 patients and two carriers in 281 controls in the ADNI cohort (gene-based *P* = 0.29, OR = 2.886), suggesting a higher frequency of *C7* mutations in European patients [54], albeit the pattern might be different from that in Han Chinese. Among them, rs3792646-C (p.K420Q) and chr5:40936541 C>T (p.C128R) occurred one and five times, respectively, in 296 patients with Alzheimer's disease or late-stage mild cognitive impairment, whereas in the 281 controls, no individual harbored p.K420Q and only one individual with p.C128R was found. Although there seemed to be a trend of *C7* mutation in patients, the enrichment was not significant (p.K420Q, *P* = 0.33; p.C128R, *P* = 0.20). We also retrieved the summary statistics of the International Genomics of Alzheimer's Project [18], a large GWAS meta-analysis of Alzheimer's disease (17 008 cases versus 37 154 controls), to investigate the association between *C7* variants and Alzheimer's disease in Europeans. No nominally significant *C7* SNPs were observed. In the recently released Alzheimer's Disease Sequencing Project (ADSP) cohort [55], there were also no significant exonic variants in *C7* that showed an association with Alzheimer's disease. Only one p.K420Q carrier was found in the ADSP cohort [55], which contains 10 570 individuals of European ancestry. These results were consistent with the low allele frequency of rs3792646 in the non-Chinese populations from ExAC [50]: 0.0020 in 5041 Ashkenazi Jews, 0.0020 in 14 972 South Asians, 0.00093 in 16 649 Latinos, 0.00034 in 62 858 non-Finnish Europeans, 0.00013 in 11 946 Africans and 0.000039 in the Finnish population (http://gnomad.broadinstitute.org/variant/5–40955653-A-C, accessed on February 8 2018), suggesting that this variant is most likely Chinese- or East Asian-specific.

### Association of rs3792646 with Alzheimer's-related endophenotypes and preclinical impairments

**Figure 2.**
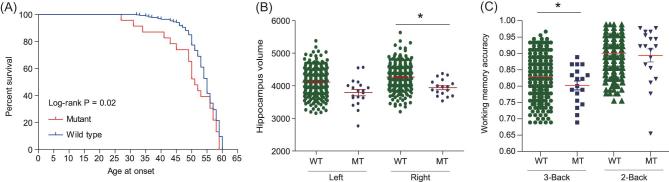
Clinical effects of *C7* rs3792646 (p.K420Q). (A) Effect of rs3792646 on age at onset (AAO) in patients with early-onset Alzheimer's disease. Carriers of C7 mutant p.K420Q had a younger AAO relative to carriers of wild type C7. (B and C) Carriers of rs3792646-C (p.K420Q, MT [genotypes CC+AC]) have a decreased hippocampus volume and poor working memory relative to the wild type carriers (rs3792646-A, WT [genotype AA]). Working memory test was performed for two-back and three-back tasks. Mean ± SD are shown. *, *P* < 0.05.

In addition to its effect on disease risk, we investigated whether rs3792646 affects the age of disease onset in our combined Han Chinese samples with available AAO information. The survival test showed that carriers of the risk allele rs3792646-C had a significant (log-rank test, *P* = 2.04 × 10^−2^) earlier onset age (51 years) than carriers of rs3792646-AA (55 years) in Han Chinese patients with an AAO < 60 years (Fig. 2A). No significant difference in the AAO was observed in patients with late-onset sporadic Alzheimer's disease.

In order to discern whether the risk allele rs3792646-C would have a potential effect on brain structure and the function of susceptible individuals in early adulthood, we took advantage of the imaging data that were previously collected in 360 healthy university students [32,56], and analyzed the association of rs3792646 with brain structural changes and working memory performance. Intriguingly, the rs3792646-C carriers (genotypes CC and AC) showed significantly lower right hippocampal volume (*P* = 0.02) and worse working memory performance (*P* = 0.03) compared with the AA carriers (Fig. 2B and C). These observations indicated that the *C7* variant rs3792646 might affect the brain function of at-risk Han Chinese individuals several decades before disease onset.

The effects of *C7* variants on Alzheimer-related endophenotypes were further investigated using the ADNI data [54]. We observed a lower hippocampal volume in only one p.K420Q carrier in the ADNI cohort [54] (Supplementary Fig. S2). Though the association between the disease and *C7* SNPs was not established in the population of European origin, two *C7* variants (Supplementary Table S3 and Fig. S2) did affect the cerebrospinal fluid Aβ and p-tau levels in the ADNI cohort [54]. In particular, carriers of p.C128R had a higher phosphorylated tau level in the cerebrospinal fluid (Supplementary Fig. S2), supporting the risk-promoting effect of *C7* variants in Alzheimer's disease.

### Upregulation of *C7* mRNA expression in brain tissues of Alzheimer's disease

**Figure 3.**
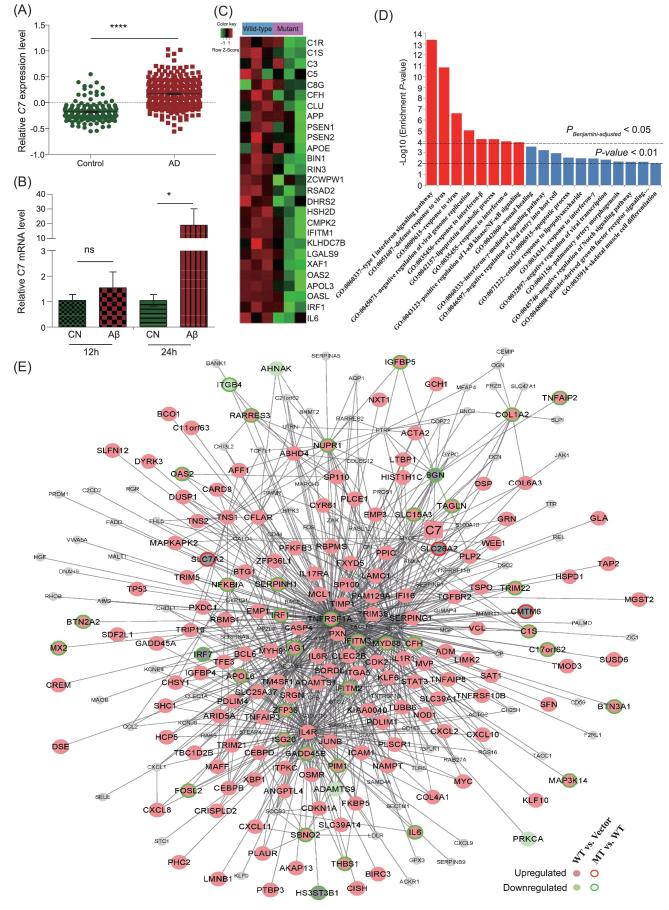
mRNA expression level changes of *C7* in brains of Alzheimer's patients, and the effects of overexpressing C7 wild type and mutant on global gene expression patterns. (A) Increased level of *C7* mRNA in frontal cortex of patients and controls based on GSE33000 [58]. ****, *P* < 0.0001. (B) Increased level of *C7* mRNA in Aβ-treated cells. U251 cells were treated with 5 μM Aβ_42_ for 12 h or 24 h before harvest for real-time quantitative PCR. Aβ was dissolved in 5% DMSO; CN, 5% DMSO. *, *P* < 0.05; ns, not significant. (C) Heatmap of the complement genes, Alzheimer's core genes and top differentially expressed genes (DEGs) upregulated in cells overexpressing wild type C7 (Wild type) but downregulated in cells overexpressing mutant p.K420Q (Mutant). (D) Enrichment of genes upregulated in cells overexpressing wild type C7 (WT, compared with vector) but downregulated in cells overexpressing mutant p.K420Q (MT, compared with WT) in biological processes determined by the gene ontology terms. DEGs were detected by RNA-seq of U251-APP cells overexpressing wild type and mutant C7. Red, Benjamini-adjusted enrichment *P*-value < 0.05; blue, original enrichment *P*-value < 0.01. (E) Enrichment of DEGs in response to C7 overexpression in the C7-involved co-expression network. The C7-involved co-expression network (immune module) was dysregulated in Alzheimer's brain tissues according to our recent gene profiling analysis for patients [52]. Significance of the enrichment of DEGs in response to C7 wild type (WT, enrichment *P* = 2.57 × 10^−6^) or mutant (MT, enrichment *P* = 2.87 × 10^−10^) overexpression in the network was measured by Fisher's exact test.

C7 is a component of the terminal complement cascade and physically interacts with the GWAS hit Clusterin (CLU) [57]. To characterize the involvement of *C7* in Alzheimer's disease, we analyzed the mRNA expression pattern of the complement cascades in frontal cortex tissues from patients and controls based on dataset GSE33000 [58]. All initial (e.g. *C1QA*, *P* = 1.8 × 10^−18^) and central (e.g. *C3*, *P* = 4.01 × 10^−9^) components of the complement cascades were significantly upregulated, whereas only *C7* was significantly upregulated of the terminal complement components in patients (*P* = 3.21 × 10^−15^, log2 fold change = 0.242) (Supplementary Table S4 and Fig. 3A). Consistently, we observed an early increase and a strong positive correlation of *C1q* and *C3* mRNA expression level with the severity of pathological changes (Aβ plaques and tau tangles) in the hippocampus of Alzheimer's disease mouse models based on the Mouseac database (www.mouseac.org) [59] (Supplementary Fig. S3; *C7* was unfortunately not included in this dataset). The increase of the *C7* mRNA level in patients could be mimicked by the significantly increased level of *C7* mRNA in U251 cells in response to Aβ treatment (Fig. 3B). All these results are consistent with recent reports that the initial complement components play an essential role in early synapse loss during the course of the development of Alzheimer's disease [60,61].

### Overexpression of C7 mutant p.K420Q disturbs the global gene expression pattern and affects cellular function

Previous studies showed that the complement components mainly function in glia [61], and that astrocytes can produce C7 and other complement components [62]. Thus, we conducted cellular analyses using the U251 glioma cell line (of astrocyte origin) and the human microglia (HM) cell line, to understand the potential biological and physiological significance of the identified risk gene *C7*. The U251 cells were engineered to stably express mutant APP K670N/M671L (U251-APP) so that they would produce Aβ_42_ under doxorubicin induction [32,37]. We performed RNA-sequencing (RNA-seq) of U251-APP cells overexpressing wild type and mutant C7 p.K420Q to determine the potential effect of the mutant. Consistent with the expression pattern of the complements in brain tissues of Alzheimer's patients (Supplementary Table S4), we observed no significant alterations in the mRNA levels of the terminal components (e.g. *C6*, *C8* and *C9*) in cells overexpressing wild type or mutant C7. The mRNA expression levels of initial components (e.g. *C1R*, *C1S* and *C3*) and regulatory factors (e.g. *C1INH* and *CFH*) of the complement cascade were significantly increased in cells overexpressing wild type or mutant C7 relative to cells transfected with empty vector (Fig. 3C). While the mRNA levels of these initial components and regulatory factors did not differ between cells overexpressing wild type C7 and mutant C7, most of the other genes (591/653) upregulated in cells overexpressing wild type C7 were downregulated in cells overexpressing mutant p.K420Q. These altered genes were significantly (*P_adj_* < 0.05) enriched in interferon-mediated signaling pathways (Fig. 3D), among which there were three GWAS-reported Alzheimer's risk genes—*BIN1*, *RIN3* and *ZCWPW1* [18]—as well as several important immune genes such as *OASL*, *IL6* and complement components (Fig. 3C). Intriguingly, the differentially expressed genes in response to C7 wild type (enrichment *P* = 2.57 × 10^−6^) or mutant (enrichment *P* = 2.87 × 10^−10^) overexpression were significantly enriched in a C7-involved co-expression network/module (Fig. 3E) that was recently recognized to be dysregulated in brains of Alzheimer's patients [52].

We further characterized the downstream effect of overexpression of the C7 mutant p.K420Q on Aβ internalization and cell apoptosis, and observed a significant impact on the internalization of fluorescently labeled Aβ_42_ in HM cells (Supplementary Fig. S4). Additionally, HM cells overexpressing mutant p.K420Q showed increased apoptosis in response to tumor necrosis factor α (TNF-α) treatment compared with cells overexpressing wild type C7 (Supplementary Fig. S5).

### Overexpression of C7 mutant p.K420Q affects excitatory synaptic transmission

**Figure 4.**
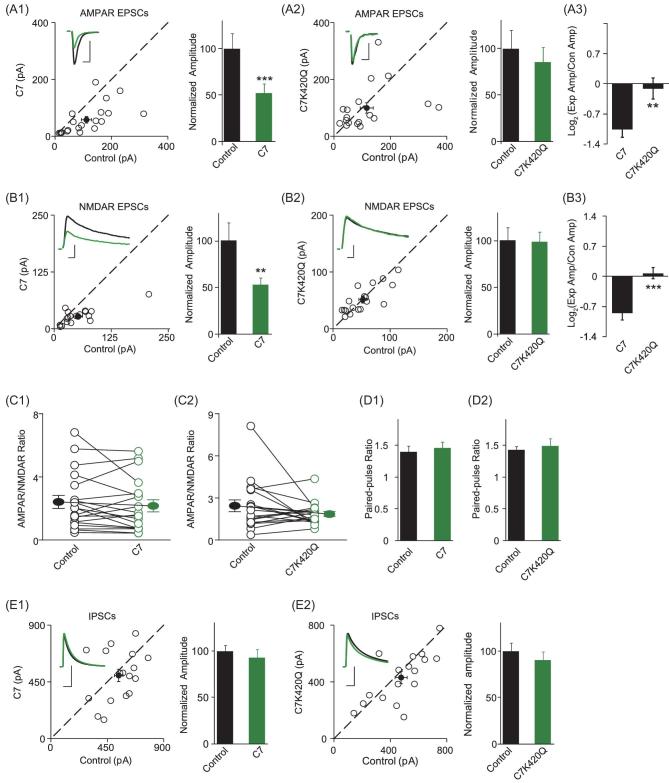
*C7* mutant p.K420Q reverses its physiological regulation of excitatory synaptic transmission. (A and B) Rat hippocampal slice cultures were biolistically transfected with wild type C7 or C7 p.K420Q. Simultaneous dual whole-cell recordings from a transfected CA1 pyramidal neuron (green trace) and a neighboring wild type one (black trace) were performed. The evoked AMPA (A1 and A2) and NMDA (B1 and B2) EPSCs were measured at –70 mV and +40 mV (the current amplitudes were measured 100 ms after stimulation), respectively. Open and filled circles represent amplitudes for single pairs and mean ± SEM, respectively. Insets show sample current traces from control (black) and experimental (green) cells. Bar graphs show normalized EPSC amplitudes (mean ± SEM) of –70 mV (A1, n = 20, 51.90 ± 10.45% control, *** *P* < 0.001; A2, n = 18, 85.43 ± 15.72% control, *P* > 0.05) and +40 mV (B1, n = 19, 52.97 ± 7.33% control, ** *P* < 0.005; B2, 98.37 ± 10.84% control, *P* > 0.05) presented in scatter plots. The scale bars for representative EPSC traces are: 100 pA/25 ms (A1) and 50 pA/25 ms (A2, B1 and B2). All the statistical analyses were compared to respective control neurons with a two-tailed Wilcoxon signed-rank sum test. (A3 and B3) Comparison of the logarithm of AMPA EPSC (A3: C7, –1.07 ± 0.19; C7 p.K420Q, –0.12 ± 0.24, ** *P* < 0.01) and NMDA EPSC (B3: C7, –0.86 ± 0.16, C7 p.K420Q, –0.07 ± 0.13, *** *P* < 0.0005) amplitude ratios between the experimental and respective control neurons (mean ± SEM) from wild type C7 and C7 p.K420Q transfections. All statistical analyses were tested using the Mann–Whitney *U*-test. (C) AMPA/NMDA ratios recorded from wild type C7 (*P* > 0.05, n = 19) or C7 p.K420Q (*P* > 0.05, n = 18) overexpression neurons are not significantly different from respective wild type ones. A two-tailed Wilcoxon signed-rank sum test is used for statistical analyses. (D) No change in paired-pulse ratio, defined as second EPSC over first EPSC, from wild type C7 (control: 1.39 ± 0.09, C7: 1.46 ± 0.09; *P* > 0.05, n = 18) or C7 p.K420Q (control: 1.43 ± 0.05, C7 p.K420Q: 1.49 ± 0.11; *P* > 0.05, n = 18) transfections. (E) Wild type C7 and C7 mutant p.K420Q have no effect on inhibitory synaptic transmission. The same experiments as in Fig. 4A except that IPSCs were measured at 0 mV. Bar graphs show normalized IPSC amplitudes (mean ± SEM) (E1, n = 17, 92.67 ± 9.08% control, *P* > 0.05; E2, n = 17, 90.43 ± 8.99% control, *P* > 0.05) presented in scatter plots. The scale bars for representative IPSC trace were: 200 pA/25 ms (E1) and 300 pA/25 ms (E2). All the statistical analyses are compared to respective control neurons with a two-tailed Wilcoxon signed-rank sum test.

Besides functions in immune activation, Aβ in-ternalization and cell apoptosis, the complement system also plays a role in neuronal activity [60,61,63–67]. Biolistic transfection on rat hippocampal slice cultures and accompanying dual whole-cell recording analyses offered a convenient study system to characterize the physiological function of target gene(s) in neurons [68]. We used this strategy to investigate the effect of C7 and its p.K420Q mutant on synaptic transmission in excitatory neurons. We found that overexpression of wild type C7 in CA1 pyramidal neurons decreased both the AMPAR (α-amino-3-hydroxy-5-methyl-4-isoxazolepropionic acid receptor) and NMDAR (N-methyl-D-aspartate receptor)-mediated synaptic transmission compared with the respective neighboring control neurons (Fig. 4A1 and B1), but that these inhibitory effects were compromised by the p.K420Q mutation (Fig. 4A2, A3, B2 and B3). However, overexpression of C7 or its mutant had no effect on the ratio of AMPAR- and NMDAR-mediated evoked excitatory postsynaptic currents (EPSCs) (Fig. 4C1 and C2), suggesting a general postsynaptic role of C7 in excitatory synaptic transmission. The paired-pulse ratio, which is the parameter for presynaptic release probability, was not affected by C7 or its mutant overexpression (Fig. 4D1 and D2). This observation indicated that the regulatory function of C7 is postsynaptic-specific. Moreover, neither wild type C7 nor C7 mutant p.K420Q had any effect on γ-aminobutyric acid (GABA) receptor-mediated inhibitory postsynaptic transmission (Fig. 4E1 and E2), indicating that the C7-mediated effect is specific to excitatory synapses. Taken together, C7 likely inhibits excitatory synaptic transmission in pyramidal neurons while the mutant p.K420Q impairs this negative regulation. Note that complement factors can be produced and secreted locally in the brain; it is surprising that neighboring non-transfected cells are not regulated by the overexpressed C7. Where endogenous and overexpressed C7 are located and what their extracellular levels are in the growing medium remain to be determined.

## DISCUSSION

To date, most of the Alzheimer's risk genes identified by GWASs and next-generation sequencing technologies have been found in populations of European ancestry [5,18,23,24,28,69]. For East Asians, there has been only one GWAS in a Japanese population, with no genome-wide significant loci (excluding *APOE*) being reported [70]. Given the increasing burden of Alzheimer's disease and the population heterogeneity, there is an urgent need to investigate the genetic basis of the disease in the Han Chinese, the largest population in the world with the greatest number of Alzheimer's patients worldwide [33,34]. In this study, we have used WES to identify potential risk gene(s) of Alzheimer's disease in Han Chinese. By recruiting relatively homogeneous set of patients with features attributable to genetic factors (familial and extreme early onset), we have countered the limitation of a small sample size and discovered a novel exome-wide significant variant rs3792646 (p.K420Q) in the *C7* gene (Fig. 1). Importantly, this risk variant has a comparable effect size with the well-known hits *TREM2* p.R47H [23,24] and *PLD3* p.V232M [25], which were identified in populations of European origin. Intriguingly, the effect size of rs3792646 (OR = 3.651, Supplementary Table S2) was dramatically increased in the *APOE* ε4 carriers, suggesting a potential interaction between this rare missense variant and *APOE* ε4.

The complement system has complex roles in Alzheimer's disease, including Aβ clearance, microglia activation, neuroinflammation, apoptosis and neuron death [60,61,63–67]. Whether or not the complement system is a driving factor or a byproduct has been a controversial topic [60]. Recent studies reported that the initial component *C1q* and the central component *C3* contribute to early synapse loss in response to Aβ and/or viral infection in Alzheimer's disease [60,61]. Our current results indicated that *C7*, a canonical terminal component in the complement cascade, might be also involved in the early pathological stage of Alzheimer's disease, together with the other initial components. Previous results have suggested that *C7* plays a major role in the formation of the membrane attack complex and that it serves as a membrane anchor [71]. *C7* deficiency contributes to susceptibility to a variety of immune and infectious diseases, such as meningococcal infection [72–75], and rare damaging variants of other complement components have been reported to be enriched in age-related macular degeneration [76]. It is also known that both infection and metabolite (e.g. Aβ) accumulation can activate the complement cascade [60,61]. While, to our knowledge, no report has linked *C7* with neurodegenerative disorders to date, our results indicate that *C7* might function in the early activation phase, rather than in the terminal membrane attack complex as previously reported [71]. Moreover, the *C7* risk allele affects the brain’s morphological structure and impairs working memory in young adults and disease-related endophenotypes in patients (Fig. 2). These results were further supported by the observation that overexpression of mutant C7 affects the global gene expression pattern (Fig. 3), Aβ internalization and apoptosis (Supplementary Figs S4 and S5), which would play an active role in the pathogenesis of Alzheimer's disease. Through the use of an electrophysiological assay with rat hippocampal slice cultures and dual whole-cell recordings, we showed that overexpression of C7 mutant p.K420Q affects the excitatory synaptic transmission of neurons (Fig. 4). All these lines of evidence suggest a putative role of *C7* and its variant in the development of Alzheimer's disease, though the exact mechanism remains to be elucidated. Considering the complex roles of the complement system in Alzheimer's disease, it is still unclear whether there is a link or interaction between C7-induced changes in glial activity and changes in synaptic function. The exact mechanism of complement genes in the disease remains to be elucidated.

Consistent with the functional assays, *in silico* prediction by four algorithms showed that the mutation p.K420Q was deleterious. Nevertheless, we should note that rs3792646 was also present in the general population (with an allele frequency ranging from 0.0004–0.03), leading to an argument against its pathogenic status, similar to the case of NR1H3 p.R415Q in multiple sclerosis [77], although the situation in Alzheimer's disease might be a bit different, partly due to its late age-of-onset. We have previously shown that a common missense variant in another complement gene, *CFH*, conferred genetic risk to Alzheimer's disease, whereas this variant underwent pathogen-driven selection so that it was retained in the population due to the trade-off effect [32]. It is reasonable to speculate that mutant C7 might also have been positively selected during evolution, and that this has led to the observed differences in allele frequencies and distinct disease susceptibility patterns.

The current study has some limitations. First, although we observed an exome-wide significant association of rs3792646 with Alzheimer's disease in the WES discovery stage and validated the association in independent cohorts, it should be noted that the association of rs3792646 in the initial screening stage did not reach exome-wide significance in the logistic regression analysis. This might have been caused, at least partially, by the small sample size in this stage (Table 1). As the association was initially recognized in early-onset patients that were selected by extreme phenotype sampling in the discovery stage, whereas most of the replication samples were sporadic late-onset patients, independent replication in larger cohorts with early-onset Alzheimer's disease is needed to further confirm the association. Second, the risk allele rs3792646-C was mainly found in Asian populations and was infrequent in European populations. In the ADNI [54] and ADSP samples [55] of European ancestry, we observed only one risk allele carrier out of 812 individuals and one carrier out of 10 570 individuals, respectively, suggesting a Chinese-specific effect of rs3792646. However, the results need further validation and should be interpreted with caution, as the sample size of cases of European ancestry is still limited. Nonetheless, our functional characterization indicates that the C7 mutant p.K420Q affects the expression of the interferon-mediated signaling pathways, Aβ internalization and apoptosis at the cellular level, as well as the excitatory synaptic transmission of neurons, which reinforces the conclusion that *C7* is a risk gene for Alzheimer's disease.

During the preparation of this manuscript, we noticed a recent publication about a whole-genome sequencing-based GWAS in a Chinese population [78]. These authors identified two common variants, *GCH1* (rs72713460) and *KCNJ15* (rs928771), showing nominal associations with Alzheimer's disease in Chinese patients. We checked these two risk variants in our WES data, but failed to find any association between these genes/variants and Alzheimer's disease in our samples. This might be caused by different strategies that were used in Zhou *et al.*’s study (low-coverage whole-genome sequencing for sporadic patients) [78] and our study (WES for a relatively homogeneous set of patients with an extreme phenotype). Evidently, large sample sizes are needed for further validation of these risk genes in our current study and the study by Zhou *et al.* [78].

## Conclusion

In summary, we have identified a rare damaging variant, rs3792646 (p.K420Q) in *C7*, which confers risk of developing Alzheimer's disease, through exome-wide screening in Chinese patients with early-onset Alzheimer's disease or familial history. Although *TREM2* p.R47H and *PLD3* p.V232M are extremely rare or absent in Chinese patients [26,31], we have shown here that the Han Chinese population harbors another risk factor, *C7* p.K420Q, with a comparable effect size. The *C7* risk allele is most likely specific to Han Chinese. This variant could potentially contribute to the risk of Alzheimer's disease via disrupting immune activation and Aβ processing, and is associated with changes in brain structure and function even decades before disease onset. Our results strongly suggest the active roles of *C7*, together with other complement components such as the GWAS hits *CR1*, *CLU* [18] and *CFH* [32], in the development of Alzheimer's disease. Further validation and functional investigation is needed to characterize the mechanisms underlying the risk for Alzheimer's disease conferred by these molecules.

## MATERIALS AND METHODS

### Subjects: extreme phenotype sampling for exome sequencing

We employed an extreme phenotype sampling strategy in the WES stage to increase the likelihood of identifying true disease-related variants [35,36]. The criteria of extreme phenotypes were set as follows [79]: (i) AAO of Alzheimer's disease ≤ 55 and/or (ii) with a positive familial history. In our collection of over 1000 genetically unrelated patients from East and Southwest China [26,32,37–41], 107 unrelated patients (46.7% females; age 64.6 ± 10.29 years; AAO 56.0 ± 9.83; *APOE* ε4, 38.5%) met the criteria and were subjected to WES. For familial Alzheimer's disease, only the probands were included in the study and no family members were recruited. Detailed clinical records including age, sex, education, occupation, AAO, familial history, disease history, diagnostic imaging tests and neuropsychological assessment were collected for each participant. All patients were diagnosed by at least two clinical psychiatrists using the revised National Institute of Neurological and Communicative Disorders and Stroke and the Alzheimer's Disease and Related Disorders Association criteria [80,81] and the Diagnostic and Statistical Manual of Mental Disorders, Fourth Edition, as described in our previous studies [26,32,37–41]. In total, 160 in-house control individuals (40.6% females, age 52.6 ± 16.5 years; *APOE* ε4, 15%) [42], showing no signs of memory loss and without any familial history of neurodegenerative disorders, were compared with the patients with Alzheimer's disease. Sample collection complied with the Declaration of Helsinki, with written informed consent being obtained from each participant or their guardian. This study was approved by the Institutional Review Board of Kunming Institute of Zoology, Chinese Academy of Sciences.

### WES and data processing

The coding region (untranslated regions and exons, namely exome) of the whole genome of cases and in-house controls was captured using the SeqCap EZ Exome Kit v3.0 (#06465692001, Roche, Basel, Switzerland). The total size of the regions covered by 2.1 million long oligonucleotide probes was 64 Mb, achieving the most comprehensive coverage of coding regions in the genome. All the genome coordinates were based on human genome build GRCh37 (hg19, http://asia.ensembl.org/info/website/tutorials/grch37.html). Processed final libraries were pooled and sequenced on an Illumina HiSeq2500 or 4000 (150-bp paired-end, Illumina, San Diego, CA, USA).

Low-quality raw reads were removed using Trimmomatic-0.32 [82] with the parameters ‘LEADING:3 TRAILING:3 SLIDINGWINDOW:4:15 MINLEN:36’. Quality-filtered reads were then aligned to the National Center for Biotechnology Information human genome reference assembly (build GRCh37) using the Burrows–Wheeler Aligner [83]. Picard Tools (http://broadinstitute.github.io/picard/) were used to flag duplicate reads. Variant calling was performed through the canonical pipeline recommended by the Best Practice Variant Detection with the GATK (Genome Analysis Toolkit) [84]. Variant Quality Score Recalibration from the GATK package was used to filter spurious variants resulting from sequencing errors and mapping artifacts. ANNOVAR was used to annotate variants into different functional categories according to their locations and expected effects on encoded gene products [85].

In order to achieve credible statistical power by increasing the control:case ratio, we pooled the exome data of the 160 in-house non-dementia individuals [42] with the whole-genome data of Han Chinese in Beijing (n = 103) and Southern Han Chinese (n = 105) from phase 3 of the 1000 Genomes Project [43] as the initial population control (n = 368). PC analysis was performed to ensure that there was no apparent population stratification between the studied subjects and the reference Chinese populations from phase 3 of the 1000 Genomes Project [43] by using the GCTA tool (http://cnsgenomics.com/software/gcta/#Overview). Based on the clustering pattern (Supplementary Fig. S1), there is no obvious population substructure among the East Asian populations, suggesting that it is reasonable to group the in-house controls with Han Chinese in Beijing and Southern Han Chinese from phase 3 of the 1000 Genomes Project [43] as the general population control. Allele frequencies of exonic variants in patients were compared to that of the population controls by using the Fisher's exact test in the initial exome-wide case-control screen. To rule out the possibility of technical artifacts due to potential population substratification, we performed a logistic regression with PC1–3 as the covariates using the open-source C/C++ toolset Plink/seq (https://atgu.mgh.harvard.edu/plinkseq/). We also included *APOE* ε4 status and sex as covariates, besides PC1–3, in the logistic regression analysis.

We defined nonsense, frameshift, splice site and missense variants as functional if these variants were predicted to be damaging by at least one of the five algorithms (PolyPhen2 HumDiv and HumVar [44], LRT [45], MutationTaster [46] and SIFT [47,48]). Functional variants with a MAF < 5% in the 368 pooled population controls [42,43] were analyzed to identify the exome-wide significant rare variants. A total of 23 373 functional variants met this criterion, resulting in a threshold for the exome-wide significance of *P* < 2.139 × 10^−6^ (Bonferroni corrected: 0.05/23373). These exonic variants were directly compared to the population control by using Fisher's exact test and logistic regression analysis. All damaging missense variants with a MAF < 5% in the control population were used for the gene-based burden testing [86] using PLINK/seq. The SKAT was also used to evaluate the combined effect of rare mutations using the SKAT R package [53]. Allele frequencies of the targeted loci in 4327 East Asians from ExAC [50] and in 11 670 Chinese samples from the CONVERGE Consortium (the largest Han Chinese low-coverage genome dataset so far) [51] were retrieved and used as the reference control for comparison with the Alzheimer's patients.

### Independent validations in Chinese and European populations

The discovery WES screen (stage 1) revealed a significant association between *C7* rs3792646 and early-onset and familial Alzheimer's disease. To validate this association, we sequenced this SNP in an independent Han Chinese sample with early-onset and/or familial Alzheimer's disease from Beijing (stage 2, n = 103 cases; 60.2% females, age 61.2 ± 7.33 years, AAO 57.2 ± 9.12 years; *APOE* ε4, 24%). We attempted to confirm the association of *C7* rs3792646 with Alzheimer's disease in independent cohorts with sporadic Alzheimer's disease (stage 3): the East China cohort contains 587 sporadic cases (61.0% females, age 79.5 ± 8.45 years, AAO 73.2 ± 9.25 years; *APOE* ε4, 41%) and 274 geographically matched controls, while the Southwest China cohort contains 583 sporadic cases (61.9% females, age 76.4 ± 10.04 years, AAO 74.7 ± 11.79 years; *APOE* ε4, 34%) and 2190 geographically matched controls. We also analyzed a patient sample from Hunan, Southcentral China (n = 235 sporadic cases; 63.9% females; age 79.1 ± 7.86 years; AAO 74.4 ± 7.72 years; *APOE* ε4, 35%). DNA fragments covering SNP rs3792646 were amplified using the primer pair 5′-TATAACGACATGTGCCCACCA-3′/5′-GACTTCAGGAGCCCACAAGC-3′ and sequenced using the primer 5′-GCCCTAAATATCCTTTGTGCT-3′.

Whole-genome sequencing data and clinical phenotypes of 812 individuals of European ancestry (including 281 controls, 483 subjects with mild cognitive impairment and 48 subject with Alzheimer's disease) were retrieved from the ADNI project (http://adni.loni.usc.edu/) [54] to explore rare *C7* variants in Europeans. Given the small sample size of Alzheimer's patients in ADNI data, patients with late-stage mild cognitive impairment were combined with Alzheimer's patients to achieve better statistical power, resulting in 296 patients and 281 controls (named ‘ADNI cohort’ in the text), whereas the remaining 235 subjects with early-stage or modest mild cognitive impairment were excluded from the analysis [54]. To validate the result in a larger European cohort, we obtained access to the WES data of 5815 Alzheimer's cases and 4755 controls from the ADSP [55] through the dbGaP (Genotypes and Phenotypes database) under the study accession phs000572.v7.p4 (accessed in May 2018).

Statistical power and sample size calculations were performed using Quanto software (version 1.2.4) [87] based on the observed parameters. For alleles with a MAF of 0.05 in the general population (disease prevalence was set as 0.1), at least 279 pairs of case and control samples were needed to capture an OR of 2.0 with a statistical power of 80% under an additive model. The current samples thus had sufficient power for validating associations with considerable effect sizes.

### Brain structural changes and cognitive performance of at-risk individuals in early adulthood

We had previously recruited 360 young healthy adults (48% females, age 19.4 ± 1.1 years) to study the effects of potentially functional variants on morphological and functional changes of the brain [32,56]. All these participants were university students without any history of neuropsychiatric disorders or acquired brain injury. Their brain structure data were collected through structural magnetic resonance imaging scans using an MR750 3.0 Tesla magnetic resonance scanner (GE Healthcare). Briefly, a high-resolution 3D T1-weighted brain volume (BRAVO) sequence was performed with the following parameters: repetition time (TR) = 8.16 ms, echo time (TE) = 3.18 ms, flip angle = 7°, field of view (FOV) = 256 mm × 256 mm, voxel size = 1 × 1 × 1 mm^3^ and 188 slices. The brain regions of interest were the hippocampus and entorhinal cortex, which were recognized as the brain regions most and the first affected by Alzheimer's disease, respectively [88,89]. The magnetic resonance imaging data were analyzed with FreeSurfer software (version 5.3) [90] as previously described [32,56,91]. These young healthy donors also received a working memory test during their participation [92]. The working memory task was assessed with an N-back paradigm (two- and three-back) [93]. In brief, participants were presented with a series of letters sequentially, and were asked to perform continuous judgments: whether the letter on the screen was the same as the one presented two letters earlier (two-back task) or the one presented three letters earlier (three-back task) [92]. We excluded the outliers in accuracy (more than mean + 2 SD or lower than mean – 2 SD) in the analysis of group differences in working memory performance. The Alzheimer's disease-related variant rs3792646 was genotyped in these healthy donors by sequencing as described above, and the effects of rs3792646 genotypes on morphological changes and working memory performance were assessed.

### Effects of rs3792646 genotypes on Alzheimer-related endophenotypes

In order to further investigate the role of rs3792646 in the pathogenesis of Alzheimer's disease, we obtained genetic, neuroimaging and biomarker data from 812 individuals in the ADNI dataset [54]. The primary goal of the ADNI has been to test whether serial magnetic resonance imaging, positron emission tomography and other biological markers, as well as clinical and neuropsychological assessment, can be combined to measure the progression of mild cognitive impairment and the early stages of Alzheimer's disease [54]. The effects of disease-risk SNPs on endophenotypes, e.g. the levels of tau, p-tau and Aβ in the cerebrospinal fluid, cognitive score and hippocampus volume, were analyzed using PLINK [94].

### Cell culture and transfection

U251 glioma cells and HM cells were introduced from Kunming Cell Bank, Kunming Institute of Zoology, Chinese Academy of Sciences. The U251 cells were engineered to stably express mutant APP K670N/M671L (U251-APP) so that they would produce Aβ_42_ under doxorubicin induction [32,37]. We overexpressed wild type and mutant p.K420Q of C7 in these two cell lines to characterize their potential roles. Briefly, U251-APP cells were cultured in Roswell RPMI-1640 medium (HyClone, #C11875500BT) supplemented with 10% fetal bovine serum (Gibco-BRL; #10099–141), 100 U/ml penicillin and 100 mg/ml streptomycin in 5% CO_2_ at 37°C. HM cells were maintained in Dulbecco's modified Eagle's medium (Gibco-BRL; #11965–092) supplemented with 10% fetal bovine serum (Gibco-BRL; #10099–141). Transfection of empty vector (pReceiver-M14 [Cytomegalovirus promoter, 3 × Flag], GeneCopoeia, Inc.), or C7 wild type and mutant p.K420Q expression vectors, was performed using an electroporator (CUY21EDIT, Nepa gene Co., Japan) following the manufacturer's instructions. In brief, cells were trypsinized and washed three times with Opti-MEM medium (Gibco-BRL). Around 1 × 10^6^ cells were resuspended in 100 μl Opti-MEM medium, and electroporated with 10 μg plasmids. Transfected cells were seeded in prewarmed growth medium for 72 h in 5% CO_2_ at 37°C before being harvested.

### RNA-seq and mRNA expression profiling

We performed transcriptome sequencing for U251-APP cells overexpressing wild type or mutant C7 protein. After RNA quantification and qualification, 1.5 μg RNA per sample was used for the library preparation. Sequencing libraries were generated using a NEBNext Ultra^TM^ RNA Library Prep kit for Illumina (New England Biosciences, USA) following the manufacturer's recommendations. Index codes were added to attribute sequences to each sample. The processed final library was sequenced on an Illumina Hiseq 4000 platform and 150 bp paired-end reads were generated. Sequenced reads were processed and differential gene expression analysis was performed according to standard protocols. In brief, the raw reads were trimmed to remove sequencing adapters and low-quality reads. The clean reads were then aligned to the reference genome (hg19) using Tophat [95]. HTSeq-count [96] was then used to count aligned reads that mapped to the annotated human genes (gencode v19) [97]. Gene-level differential expression analyses were performed using DESeq2 [98]. PC analysis of gene expression levels was performed to remove outliers using the ‘prcomp’ function in the ‘stats’ package in R (http://www.R-project.org/). Hierarchical cluster analyses and heatmap analyses were performed using R-statistics. Gene ontology biological processes enrichment analysis for differentially expressed genes was performed using the Database for Annotation, Visualization and Integrated Discovery (DAVID) online tools (https://david.ncifcrf.gov/) [99]. The global effects of C7 wild type and mutant overexpression were assessed using the co-expression network that was constructed based on expression profiles of brain tissues from individuals with Alzheimer's disease [52]. The network was visualized using Cytoscape software [100].

We retrieved GSE33000 from the GEO (Gene Expression Omnibus, https://www.ncbi.nlm.nih.gov/geo/browse/) database, a microarray expression profile of frontal cortex from 309 Alzheimer's patients and 156 controls [58], to re-analyze the expression pattern of the complement components in brains of Alzheimer's patients. Differential gene expression analysis was performed using linear regression with the *limma* package in R, as described elsewhere [52]. In addition to the differential expression analysis in brain tissues of patients, we also analyzed the expression alterations in brain tissues of mouse models [59]. In brief, the transgenic mouse models with human mutant genes responsible for familial type of Alzheimer's disease, which showed Alzheimer's pathological features such as amyloid plaques and neurofibrillary tangles, were used for genome-wide gene profiling [59]. Expression profiling of hippocampal and cortex tissues were tested using the MouseRef8 v2 (Illumina) microarray platform. Microarray data was processed and shared by John Hardy and colleagues from the Mouse Dementia Network, available at Mouseac (www.mouseac.org) [59]. More details about this dataset were described in the original paper [59]. The number of Aβ plaques and the level of tau burden were quantified. Correlations between mRNA expression of genes of interest and the quantified indices of pathology were then measured based on the processed data, using Pearson's correlation test.

### Aβ_42_ internalization and cell viability

HM cells were treated with 5 μM oligomeric, aggregated and fibrillary fluorescently-labeled Aβ_42_ (ChinaPeptides Co., Ltd.) for 2 h after transfection with the C7 wild type and mutant overexpression vectors for 24 h. Fluorescein isothiocyanate intensity in treated cells was measured by flow cytometry using an LSRFortessa cell analyzer (Becton Dickinson, USA) following the manufacturer's instructions. FlowJo software was used to view and analyze the flow cytometric data.

Cell viability induced by TNF-α (Sigma) was determined using the MTT (3-(4,5-dimethylthiazol-2-yl)-2,5-diphenyltetrazolium bromide; Sigma, #M2128) assay. HM cells were seeded in 96-well plates at a density of 5 × 10^3^ cells per well after transfection for 24 h, followed by treatment with 2 μg/ml actinomycin D (Merck Millipore, #129935) and 200 ng/ml TNF-α (peproTech, #300–01A) for 24 h. The MTT assay was then performed according to the manufacturer's instructions. Absorbance was measured at 490 nm using a Gen5 plate reader (Elx808, BioTek).

### Electrophysiology in brain slice cultures

The electrophysiology in brain slice cultures was determined in accordance with the previously described protocol [68,101]. Briefly, organotypic rat hippocampal slice cultures were made from postnatal day 6–8 wild type rats. The C7 wild type and mutant p.K420Q were subcloned into the pCAGGS vector harboring enhanced green fluorescent protein. Biolistic transfections were carried out after culture for 2 days using a Helios Gene Gun (Bio-Rad) with 1 μm DNA-coated gold particles. Slices were maintained at 34°C with media changes every other day. On day 6 after transfections, voltage-clamp dual whole-cell recordings for CA1 pyramidal neurons were taken from a fluorescently transfected neuron and a neighboring untransfected control neuron. During recording, slices were transferred to a perfusion stage on an Olympus BX51WI upright microscope and perfused at 2.5 ml/min with artificial cerebrospinal fluid bubbled with 95% O_2_/5% CO_2_. The artificial cerebrospinal fluid was composed of 119 mM NaCl, 2.5 mM KCl, 4 mM CaCl_2_, 4 mM MgSO_4_, 1 mM NaH_2_PO_4_, 26.2 mM NaHCO_3_ and 11 mM glucose. Series resistance was monitored online, and recordings in which the series increased to > 30 MOhm or varied by > 50% between neurons were discarded. Dual whole-cell recordings measuring EPSCs and inhibitory postsynaptic currents (IPSCs) were performed. When measuring EPSCs, 100 μM picrotoxin was added to block inhibitory currents and 4 μM 2-chloroadenosine was used to control epileptiform activity. When measuring IPSCs, 10 μM NBQX (AMPAR antagonist) and 50 μM D-APV (NMDAR antagonist) were added to block AMPAR and NMDAR-mediated excitatory currents, respectively. Internal solution contained 135 mM CsMeSO_4_, 8 mM NaCl, 10 mM HEPES, 0.3 mM EGTA, 5 mM QX314-Cl, 4 mM MgATP, 0.3 mM Na_3_GTP and 0.1 mM spermine. A bipolar stimulation electrode was placed in the stratum radiatum and responses were evoked at 0.2 Hz. Peak AMPAR responses were recorded at –70 mV, and NMDAR responses were recorded at +40 mV, with amplitudes measured 100 ms after stimulation to avoid contamination by the AMPAR current. The paired-pulse ratio was determined by delivering two stimuli 40 ms apart and dividing the peak response to stimulus 2 by the peak response to stimulus 1. Peak GABA currents were recorded at 0 mV. All the data were analyzed offline with custom software (Igor Pro). Responses were collected with a Multiclamp 700A amplifier (Axon Instruments), filtered at 2 kHz and digitized at 10 kHz. All animal experiments were performed in accordance with established protocols approved by the Institutional Animal Care and Use Committee of Kunming Institute of Zoology, Chinese Academy of Sciences.

### Statistical analysis

Statistical comparisons between two groups concerning relative cell numbers, imaging analysis and cognitive tests were conducted using a two-tailed Student's *t*-test. The significance of pathway or network enrichment was measured by using Fisher's exact test. The significance of evoked dual whole-cell recordings compared to controls was determined using the two-tailed Wilcoxon signed-rank sum test. For experiments involving unpaired data, a Mann–Whitney *U*-test with Bonferroni correction for multiple comparisons was performed. Paired-pulse ratios were analyzed with the Student's *t-*test. All statistical analyses were carried out with Igor Pro (Wavemetrics) and GraphPad Prism (GraphPad Software). We used the survival test to show the potential effect of *C7* variant rs3792646 on the AAO of Alzheimer's disease. In brief, Alzheimer's patients were grouped into the *C7* wild type group and mutant carrier group, and the AAO was set as the number of deaths/events. The survival proportion was assessed using the log-rank (Mantel–Cox) test with GraphPad Prism.

## DATA AVAILABILITY

The summary statistics of all 23 373 rare or low-frequency coding variants identified in stage 1 for initial discovery have been deposited into the AlzData webserver (http://www.alzdata.org/exome.html) [52] and are freely accessible. RNA-seq data of U251-APP cells overexpressing wild type C7, mutant C7 (p.K420Q) and empty vector are deposited in the GEO database under accession number GSE101608.

## Supplementary Material

Supplemental File
