# Supplementary material for: Complement C7 is a novel risk gene for Alzheimer's disease in Han Chinese
Source: Natl Sci Rev. 2018 Nov 5;6(2):257–74. doi: 10.1093/nsr/nwy127 (PMC6477931; doi:10.1093/nsr/nwy127)
Supplement: Supplemental File [file nwy127_supplemental_file.doc]

**Supplementary Figure 1.** **Principal component analysis (PCA) showed no population stratification between the studied subjects and the reference Chinese populations.** Han Chinese in Beijing (CHB) and Southern Han Chinese (CHS) from the 1000 Genome Project phase 3 were used as the reference Chinese populations. A total of 112,075 common SNPs shared by subjects undergoing whole exome sequencing in this study and the 1000 genomes were used in the PCA by using the GCTA tool ([http://cnsgenomics.com/software/gcta/#Overview](http://cnsgenomics.com/software/gcta/#Overview)). The PCA distinguished clearly the East Asian populations from the populations outside of East Asia, suggesting that these selected SNPs contain ancestry-informative markers. Based on the clustering pattern, there is no obvious population substructure among the East Asian populations. This result suggested that it is reasonable to group our in-house controls (N=160) with the two Han Chinese populations from the 1000 Genome Project (CHB, N=103; CHS, N=105) as the general population control (N = 368). Abbreviations in the principal component map: AD, Han Chinese with Alzheimer’s disease; In-house Control, non-dementia Han Chinese individuals; CHS, Southern Han Chinese; CHB, Han Chinese in Beijing, China; CDX, Chinese Dai in Xishuangbanna, China; JPT, Japanese in Tokyo, Japan; KHV, Kinh in Ho Chi Minh City, Vietnam; EAS, East Asian; EUR, European; AFR, African; AMR, Ad Mixed American; SAS, South Asian.

**Supplementary Figure 2**. **Effects of *C7* variants on endo-phenotypes in ADNI samples.** (**A**) Decreased hippocampus volume of the rs3792646-C (p.K420Q) carrier (MT, genotype AC) compared with wild type carriers (WT, genotype AA) in the ADNI sample containing 812 individuals . The effect of AD-risk SNP on hippocampus volume was analyzed by using PLINK . (**B-C**) Two rare variants of *C7* affected the cerebrospinal fluid (CSF) Aβ and p-tau levels. *, *P* < 0.05, linear regression analysis; values were shown as mean ± SD.

**Supplementary Figure 3. Expression change of *C1q* and *C3* mRNA levels in hippocampus tissues of AD mouse models.** *C1q* and *C3* mRNA expression levels increase along with the severity level of pathology (Aβ plaques [red] and tau tangles [blue]) . Expression data and pathological features of wild type and AD mouse models were downloaded from the Mouseac database (http://www.mouseac.org) . Red line, transgenic mice with homozygous human mutant APP (K670N/M671L) and PSEN1 (M146V, TPM), HO _TASTPM; Blue line, transgenic mice with human mutant MAPT (P301L), TAU; Black line, wide type mice. Data shown were mean ± SD.

.

**Supplementary Figure 4. Overexpression of C7 mutant p.K420Q affected the internalization of Aβ in human microglia (HM) cells.** HM cells were treated with 5 μM oligomeric, aggregated, and fibrillary fluorescently-labeled Aβ_42_ for 24 h after transfection of expression vectors for the C7 wild type (C7^WT^) and mutant p.K420Q (C7^MT^) and empty vector (Vector), respectively. Cells were harvested 2 h after Aβ_42_ treatment (in triples). Fluorescence intensity was measured by flow cytometry based on 10,000 cells. The FlowJo software was used for viewing and analyzing flow cytometric data. Starting cell population was determined by forward and side scatter gating, to remove debris, cell fragments, and pyknotic cells. The events with very low FSC and SSC, as well as those with very high FSC and SSC are eliminated and the major (>80%) density of events is captured by this gate (**A-B**, top panel). After identification of the cell population of interest, unstained cells (without Aβ_42_ treatment) were used as negative controls in setting the voltages and negative gates (**A-B**, bottom panel), to determine the level of background fluorescence or autofluorescence. (**C**) The mean fluorescence intensity (mean ± SD) of stained cell population for each group was compared by Student’s *t* test. *, two-tailed *P*-value < 0.05.

**Supplementary Figure 5.** **Overexpression of C7 mutant p.K420Q promoted cell apoptosis.** Cell viability induced by TNF-α was determined by using the 3-(4,5-dimethylthiazol-2-yl)-2,5-diphenyltetrazolium bromide (MTT) (Sigma, #M2128) assay. HM cells were seeded in each well of 96-well plates at a density of 5×10^3^ cells per well after transfection of expression vectors for the C7 wild type (C7^WT^), mutant p.K420Q (C7^MT^) and empty vector (Vector) for 24 h, respectively, then were treated with 2 μg/mL Actinomycin D (Merck Millipore, #129935) and 200 ng/ml TNF-α (peproTech, #300-01A). After 24 h incubation, the MTT assay was performed according to the manufacture’s instruction. Absorbance measurements were obtained by using a Gen5 plate reader (Elx808, BioTek) at 490 nm. Shown (mean ± SD) were relative values of absorbance normalized to the corresponding treatment without TNF-α treatment. Difference measured by Student’s *t* test. ***, two-tailed *P*-value < 0.001.

**Supplementary Table 1. Top 100 hits showing suggestive significant associations (*P* < 0.01) with Alzheimer’s disease in the whole exome sequencing stage**

| Chr | Position | SNP_ID | Allele | Gene | Function | AC/AN_AD | AC/AN_Ctrl | Fisher *P* | Fisher OR | PC adj *P* | PC adj OR | Sex adj *P* | Sex adj OR | APOE adj *P* | APOE adj OR | PC Sex APOE adj *P* | PC Sex APOE adj OR |
| --- | --- | --- | --- | --- | --- | --- | --- | --- | --- | --- | --- | --- | --- | --- | --- | --- | --- |
| chr19 | 45411941 | rs429358 | T/C | APOE | p.130C>R | 51/208 | 61/736 | 3.4E-09 | 3.59 | 2.0E-07 | 3.20 | 4.2E-06 | 2.87 | 1.9E-02 | 4.59 | NA | 0.11 |
| chr5 | 40955653 | rs3792646 | A/C | C7 | p.420K>Q | 17/214 | 8/736 | 1.1E-06 | 7.85 | 9.5E-05 | 5.73 | 7.3E-06 | 8.72 | 5.4E-04 | 5.11 | 9.9E-04 | 5.38 |
| chr2 | 85570857 | rs4832169 | G/A | RETSAT | p.533A>V | 26/214 | 5/160 | 2.0E-03 | 4.29 | 1.3E-01 | 3.54 | 1.1E-04 | 7.55 | 9.3E-03 | 4.03 | 9.7E-02 | 9.72 |
| chr2 | 152566961 | rs36105240 | T/C | NEB | p.305D>G | 13/214 | 9/736 | 1.9E-04 | 5.22 | 3.6E-03 | 3.78 | 1.5E-04 | 5.85 | 6.2E-04 | 4.87 | 1.9E-03 | 5.10 |
| chr11 | 102584176 | rs34009635 | A/G | MMP8 | p.436V>A | 15/212 | 13/736 | 2.5E-04 | 4.23 | 4.5E-04 | 4.31 | 1.5E-04 | 4.97 | 1.1E-04 | 5.07 | 1.2E-03 | 5.04 |
| chr2 | 182780126 | rs78774163 | G/A | SSFA2 | p.587D>N | 22/214 | 28/736 | 6.9E-04 | 2.90 | 5.8E-03 | 2.44 | 1.6E-04 | 3.34 | 5.7E-04 | 3.01 | 8.0E-03 | 2.82 |
| chr3 | 38167080 | rs117916664 | T/C | ACAA1 | p.299N>S | 14/214 | 15/736 | 2.3E-03 | 3.36 | 1.4E-02 | 2.77 | 2.8E-04 | 4.49 | 4.0E-04 | 4.22 | 5.3E-03 | 3.86 |
| chr12 | 93171826 | rs77729665 | G/A | EEA1 | p.1262R>W | 20/212 | 23/736 | 4.9E-04 | 3.23 | 9.9E-04 | 3.14 | 4.4E-04 | 3.47 | 3.2E-03 | 2.84 | 3.9E-03 | 3.47 |
| chr2 | 231406070 | rs17275036 | G/A | SP100 | p.796A>T | 16/214 | 11/526 | 8.7E-04 | 3.78 | 1.1E-02 | 3.16 | 5.3E-04 | 4.35 | 1.2E-03 | 3.96 | 2.4E-02 | 3.34 |
| chr12 | 96371782 | rs183059673 | C/T | HAL | p.532V>I | 9/212 | 3/526 | 1.1E-03 | 7.73 | 4.4E-03 | 7.75 | 6.3E-04 | 10.81 | 1.2E-02 | 5.99 | 1.3E-03 | 15.46 |
| chr9 | 104188842 | rs3739721 | C/G | ALDOB | p.207E>Q | 9/212 | 9/736 | 8.6E-03 | 3.58 | 3.8E-02 | 3.00 | 6.7E-04 | 5.33 | 1.5E-02 | 3.65 | 8.7E-03 | 5.38 |
| chr7 | 94940782 | rs13306698 | T/C | PON1 | p.160R>G | 25/212 | 36/736 | 7.1E-04 | 2.60 | 1.0E-03 | 2.64 | 8.5E-04 | 2.82 | 1.0E-03 | 2.70 | 5.2E-03 | 2.84 |
| chr16 | 4908667 | rs3747614 | A/G | UBN1 | spliceSite | 17/212 | 19/736 | 7.7E-04 | 3.29 | 2.3E-04 | 4.27 | 1.1E-03 | 3.54 | 1.8E-02 | 2.61 | 1.4E-03 | 5.35 |
| chr13 | 25831336 | rs75005059 | T/C | MTMR6 | p.365M>V | 11/212 | 8/736 | 7.5E-04 | 4.98 | 7.3E-03 | 4.22 | 1.2E-03 | 4.97 | 3.3E-03 | 4.36 | 6.8E-03 | 5.26 |
| chr17 | 80040034 | rs2228306 | A/G | FASN | p.2005V>A | 8/212 | 6/736 | 4.7E-03 | 4.77 | 1.4E-02 | 4.38 | 1.5E-03 | 6.19 | 8.9E-03 | 4.88 | 8.3E-03 | 6.56 |
| chr12 | 56351128 | rs2071024 | G/T | SILV | p.320P>H | 14/212 | 14/736 | 1.8E-03 | 3.65 | 5.2E-03 | 3.14 | 1.7E-03 | 3.66 | 1.1E-02 | 2.89 | 1.9E-01 | 1.97 |
| chr2 | 231368897 | rs6705605 | G/T | SP100 | spliceSite | 16/214 | 14/526 | 6.2E-03 | 2.96 | 4.1E-02 | 2.37 | 1.9E-03 | 3.44 | 3.6E-03 | 3.20 | 5.6E-02 | 2.58 |
| chr15 | 101606145 | rs35128996 | C/T | LRRK1 | p.1835L>F | 12/210 | 14/736 | 6.5E-03 | 3.13 | 3.4E-03 | 3.78 | 2.2E-03 | 3.80 | 2.3E-02 | 2.78 | 3.6E-03 | 5.17 |
| chr16 | 89261471 | rs149307887 | G/A | CDH15 | p.785G>R | 16/212 | 19/736 | 1.6E-03 | 3.08 | 5.1E-02 | 2.12 | 2.3E-03 | 3.31 | 1.2E-02 | 2.82 | 2.8E-01 | 1.69 |
| chr14 | 50605490 | . | G/T | SOS2 | p.933T>K | 17/212 | 3/160 | 9.8E-03 | 4.56 | 4.7E-02 | 6.82 | 2.4E-03 | 7.21 | 5.1E-03 | 6.43 | 4.3E-03 | 31.57 |
| chr20 | 61167658 | rs145416632 | C/G | C20orf166 | p.43P>R | 10/212 | 9/736 | 3.5E-03 | 4.00 | 3.1E-03 | 4.47 | 2.5E-03 | 4.61 | 3.4E-04 | 5.79 | 1.2E-03 | 6.49 |
| chr9 | 104161450 | rs61755098 | G/A | ZNF189 | p.4P>L | 19/208 | 27/736 | 2.9E-03 | 2.64 | 3.4E-02 | 2.02 | 2.6E-03 | 2.81 | 8.4E-03 | 2.55 | 3.1E-02 | 2.46 |
| chr16 | 30123523 | rs138146407 | C/A | GDPD3 | p.168R>L | 8/212 | 7/736 | 8.2E-03 | 4.08 | 3.1E-02 | 3.42 | 2.8E-03 | 5.20 | 3.8E-02 | 3.42 | 1.7E-02 | 4.82 |
| chr2 | 71649966 | rs61739715 | A/G | ZNF638 | p.1108I>V | 19/214 | 30/736 | 8.0E-03 | 2.29 | 6.4E-02 | 1.91 | 3.0E-03 | 2.81 | 1.9E-03 | 2.89 | 6.4E-02 | 2.22 |
| chr16 | 84256422 | rs4782905 | C/T | KCNG4 | p.321E>K | 15/212 | 21/736 | 7.5E-03 | 2.59 | 9.2E-02 | 1.89 | 3.0E-03 | 2.91 | 2.0E-03 | 3.04 | 1.3E-01 | 1.94 |
| chrX | 2867424 | rs138149353 | G/C | ARSE | p.259H>D | 12/212 | 13/736 | 5.3E-03 | 3.34 | 3.3E-02 | 2.38 | 3.3E-03 | 3.09 | 3.5E-03 | 3.09 | 8.3E-03 | 3.70 |
| chr19 | 10426524 | rs79442975 | G/A | FDX1L | spliceSite | 15/206 | 22/736 | 8.0E-03 | 2.55 | 2.9E-02 | 2.23 | 3.4E-03 | 2.91 | 2.0E-02 | 2.38 | 1.1E-01 | 2.03 |
| chr1 | 144931251 | rs142679243 | C/T | PDE4DIP | p.153S>N | 7/214 | 5/736 | 7.5E-03 | 4.94 | 6.3E-02 | 3.31 | 3.6E-03 | 6.09 | 3.6E-02 | 4.06 | 6.2E-02 | 4.26 |
| chr20 | 44047974 | rs80158178 | G/A | PIGT | p.178R>Q | 12/212 | 9/736 | 5.0E-04 | 4.85 | 5.9E-03 | 3.98 | 3.7E-03 | 4.18 | 2.3E-02 | 3.13 | 5.6E-02 | 3.41 |
| chr19 | 55795872 | . | A/C | BRSK1 | p.21H>P | 22/126 | 4/120 | 2.9E-04 | 6.13 | 2.1E-01 | 2.47 | 4.0E-03 | 3.77 | 1.1E-02 | 3.37 | 7.9E-02 | 10.02 |
| chr17 | 1944781 | rs200231675 | G/C | DPH1 | p.370V>L | 7/202 | 2/526 | 2.5E-03 | 9.41 | 1.2E-02 | 9.63 | 4.1E-03 | 10.76 | 1.0E-02 | 8.79 | 1.6E-02 | 18.83 |
| chr2 | 152420386 | rs147159176 | C/T | NEB | p.4476R>H | 9/214 | 7/736 | 3.3E-03 | 4.57 | 3.3E-03 | 5.09 | 4.2E-03 | 4.87 | 3.8E-03 | 5.10 | 1.3E-02 | 5.25 |
| chr8 | 28321247 | rs3735726 | C/T | FBXO16 | p.75R>Q | 8/212 | 7/736 | 8.2E-03 | 4.08 | 1.5E-02 | 4.23 | 4.2E-03 | 4.87 | 5.5E-03 | 4.62 | 1.3E-02 | 5.23 |
| chr19 | 12059699 | rs75607624 | T/G | ZNF700 | p.287F>C | 7/212 | 5/736 | 7.2E-03 | 4.99 | 1.1E-02 | 5.44 | 4.2E-03 | 5.92 | 1.4E-03 | 7.07 | 2.2E-03 | 11.21 |
| chr1 | 15428057 | rs140076587 | G/T | KAZ | p.522E>D | 10/214 | 7/736 | 1.2E-03 | 5.11 | 5.3E-02 | 2.73 | 4.5E-03 | 4.53 | 2.8E-03 | 4.90 | 1.5E-01 | 2.42 |
| chr10 | 75138691 | rs3750575 | C/T | ANXA7 | p.419R>Q | 15/212 | 19/736 | 5.1E-03 | 2.87 | 7.4E-02 | 1.86 | 4.5E-03 | 2.80 | 2.5E-01 | 1.58 | 8.0E-01 | 1.13 |
| chr11 | 130750661 | rs147010503 | G/A | SNX19 | p.872R>C | 11/212 | 11/736 | 3.7E-03 | 3.61 | 7.7E-03 | 3.18 | 5.7E-03 | 3.83 | 3.4E-03 | 3.67 | 1.6E-02 | 3.55 |
| chr1 | 167780071 | rs117021474 | C/T | ADCY10 | p.1521C>Y | 7/214 | 4/736 | 3.8E-03 | 6.19 | 9.5E-03 | 4.93 | 6.3E-03 | 6.52 | 2.3E-02 | 4.96 | 5.3E-02 | 5.00 |
| chr14 | 94546058 | rs142609376 | T/C | DDX24 | p.11K>E | 8/212 | 6/736 | 4.7E-03 | 4.77 | 1.2E-02 | 4.41 | 6.7E-03 | 5.00 | 7.5E-03 | 4.84 | 5.9E-03 | 6.14 |
| chr15 | 63893706 | rs181302627 | C/T | FBXL22 | p.183P>S | 9/206 | 9/736 | 7.4E-03 | 3.69 | 2.8E-03 | 4.91 | 6.9E-03 | 4.12 | 1.9E-02 | 3.41 | 1.8E-02 | 4.69 |
| chr9 | 88257811 | rs143779850 | T/A | AGTPBP1 | p.371E>D | 8/212 | 7/736 | 8.2E-03 | 4.08 | 8.6E-02 | 2.64 | 8.1E-03 | 4.06 | 6.4E-02 | 2.96 | 1.4E-01 | 2.90 |
| chr3 | 129811029 | rs191831656 | C/T | ALG1L2 | p.73R>W | 8/214 | 6/736 | 5.0E-03 | 4.72 | 1.4E-02 | 3.76 | 8.7E-03 | 4.07 | 1.6E-02 | 3.53 | 1.1E-02 | 4.25 |
| chr8 | 120629807 | rs148588719 | A/C | ENPP2 | spliceSite | 17/212 | 18/736 | 5.7E-04 | 3.48 | 3.4E-03 | 2.93 | 9.6E-03 | 2.96 | 1.4E-03 | 3.50 | 4.9E-02 | 2.53 |
| chr17 | 37829778 | rs142596676 | T/C | PGAP3 | p.228N>S | 11/212 | 9/736 | 1.3E-03 | 4.42 | 1.2E-02 | 3.44 | 9.8E-03 | 3.85 | 4.9E-02 | 3.01 | 5.4E-02 | 3.38 |
| chr14 | 89171861 | rs144622692 | T/C | EML5 | p.633I>V | 6/212 | 2/576 | 6.0E-03 | 8.36 | 3.5E-02 | 5.77 | 1.0E-02 | 8.96 | 1.3E-02 | 8.64 | 1.2E-01 | 4.48 |
| chr2 | 209204243 | rs148994064 | G/A | PIKFYVE | p.1592G>R | 8/212 | 4/576 | 4.3E-03 | 5.61 | 3.8E-03 | 6.86 | 1.0E-02 | 5.49 | 1.0E-02 | 5.47 | 4.2E-02 | 5.42 |
| chr19 | 36674347 | rs79279971 | T/C | ZNF565 | p.174K>R | 9/212 | 7/736 | 3.1E-03 | 4.62 | 2.6E-02 | 3.43 | 1.2E-02 | 4.22 | 6.8E-03 | 4.63 | 9.0E-02 | 3.11 |
| chr8 | 120592406 | rs2289886 | T/C | ENPP2 | p.629N>S | 16/212 | 19/736 | 1.6E-03 | 3.08 | 1.0E-02 | 2.67 | 1.3E-02 | 2.81 | 2.1E-03 | 3.36 | 5.5E-02 | 2.45 |
| chr15 | 63014548 | rs10775181 | A/G | TLN2 | spliceSite | 200/212 | 723/736 | 5.3E-03 | 0.30 | 1.1E-03 | 0.22 | 1.3E-02 | 0.31 | 6.3E-03 | 0.30 | 2.6E-02 | 0.26 |
| chr10 | 85944516 | rs76221724 | G/T | C10orf99 | p.80Q>H | 13/212 | 13/736 | 1.6E-03 | 3.63 | 8.1E-04 | 4.29 | 1.3E-02 | 3.21 | 1.4E-02 | 2.96 | 7.7E-02 | 2.70 |
| chr14 | 21790040 | rs10151259 | G/T | RPGRIP1 | p.547A>S | 6/212 | 1/576 | 2.0E-03 | 16.75 | 5.7E-03 | 21.68 | 1.5E-02 | 15.42 | 1.2E-02 | 16.92 | 9.5E-03 | 23.27 |
| chr15 | 80450501 | rs151264725 | G/T | FAH | p.61V>F | 11/212 | 11/736 | 3.7E-03 | 3.61 | 1.3E-02 | 3.09 | 1.6E-02 | 3.10 | 1.6E-02 | 3.12 | 5.0E-02 | 3.02 |
| chr3 | 170715865 | rs140138702 | G/C | SLC2A2 | p.468L>V | 10/214 | 10/736 | 5.9E-03 | 3.56 | 6.3E-03 | 4.05 | 1.6E-02 | 3.43 | 9.6E-04 | 4.87 | 1.9E-02 | 4.30 |
| chr10 | 121565909 | rs3736822 | A/G | INPP5F | p.453I>V | 10/212 | 10/736 | 5.6E-03 | 3.59 | 1.5E-03 | 5.23 | 1.6E-02 | 3.43 | 2.6E-02 | 3.11 | 1.1E-02 | 5.63 |
| chr1 | 55642119 | rs117816458 | T/A | USP24 | spliceSite | 5/214 | 1/526 | 9.0E-03 | 12.56 | 3.1E-02 | 11.18 | 1.9E-02 | 14.12 | 9.1E-03 | 18.23 | 1.8E-02 | 18.82 |
| chr19 | 49362376 | . | C/G | PLEKHA4 | p.238R>P | 15/198 | 1/160 | 1.3E-03 | 13.03 | 7.5E-02 | 18.63 | 1.9E-02 | 11.16 | 3.1E-02 | 9.37 | 6.9E-02 | 11.49 |
| chr1 | 94048138 | rs138527879 | C/T | BCAR3 | p.469R>Q | 6/214 | 2/526 | 8.9E-03 | 7.56 | 2.2E-02 | 7.78 | 2.1E-02 | 7.66 | 1.6E-02 | 7.69 | 1.7E-01 | 3.66 |
| chr10 | 17659338 | rs368871717 | T/C | PTPLA | p.1M>V | 6/106 | 3/404 | 3.5E-03 | 8.02 | 1.5E-02 | 4.86 | 2.2E-02 | 4.04 | 1.2E-01 | 2.82 | 5.3E-02 | 7.35 |
| chr1 | 976598 | rs200607541 | C/T | AGRN | p.258T>I | 7/124 | 7/508 | 9.6E-03 | 4.28 | 1.8E-02 | 3.38 | 2.3E-02 | 3.01 | 2.6E-02 | 3.01 | 7.9E-03 | 4.73 |
| chr8 | 52321722 | rs200216958 | A/C | PXDNL | p.821L>R | 5/208 | 1/576 | 6.0E-03 | 14.16 | 6.2E-02 | 8.00 | 2.3E-02 | 12.07 | 2.6E-02 | 11.89 | 2.2E-02 | 15.25 |
| chr9 | 4662580 | rs190018180 | G/A | PPAPDC2 | p.69G>S | 11/206 | 9/736 | 1.1E-03 | 4.56 | 4.9E-04 | 6.72 | 3.2E-02 | 3.21 | 5.9E-03 | 4.02 | 1.2E-02 | 5.79 |
| chr2 | 209215654 | rs137922460 | C/T | PIKFYVE | p.1865A>V | 8/214 | 7/736 | 8.6E-03 | 4.04 | 1.8E-02 | 3.91 | 3.3E-02 | 3.60 | 3.8E-03 | 5.10 | 2.7E-01 | 2.38 |
| chr1 | 36552858 | rs142743253 | G/T | TEKT2 | p.267K>N | 8/214 | 7/736 | 8.6E-03 | 4.04 | 5.2E-03 | 4.90 | 3.3E-02 | 3.60 | 7.9E-03 | 4.68 | 1.4E-02 | 5.82 |
| chr15 | 42138159 | rs144874529 | A/G | JMJD7-PLA2G4B | p.736Y>C | 5/200 | 1/576 | 5.2E-03 | 14.74 | 6.9E-02 | 6.97 | 3.4E-02 | 10.99 | 9.3E-02 | 7.29 | 7.3E-02 | 7.80 |
| chr5 | 60050522 | rs116939630 | G/A | ELOVL7 | p.259R>C | 2/212 | 35/736 | 8.2E-03 | 0.19 | 2.4E-02 | 0.18 | 4.1E-02 | 0.12 | 2.5E-02 | 0.10 | 1.0E+00 | 0.00 |
| chr8 | 30701641 | rs142485241 | C/G | TEX15 | p.1631Q>H | 2/212 | 35/736 | 8.2E-03 | 0.19 | 6.0E-02 | 0.25 | 4.2E-02 | 0.13 | 6.9E-02 | 0.26 | 1.3E-01 | 0.20 |
| chr11 | 68854029 | rs78034812 | C/T | TPCN2 | p.681S>L | 18/212 | 28/736 | 9.8E-03 | 2.35 | 2.1E-02 | 2.19 | 4.2E-02 | 2.08 | 7.0E-03 | 2.50 | 1.0E-01 | 2.05 |
| chr11 | 119156193 | rs2227988 | C/T | CBL | p.620L>F | 2/212 | 35/736 | 8.2E-03 | 0.19 | 8.1E-02 | 0.27 | 4.5E-02 | 0.13 | 5.1E-02 | 0.24 | 2.2E-01 | 0.28 |
| chr11 | 58125774 | rs55810057 | A/G | OR5B17 | p.257Y>H | 7/212 | 5/736 | 7.2E-03 | 4.99 | 9.9E-03 | 5.46 | 4.6E-02 | 3.93 | 3.7E-03 | 6.21 | 5.2E-03 | 9.84 |
| chr1 | 16725271 | rs117944955 | G/A | SPATA21 | p.467R>* | 2/214 | 35/736 | 8.1E-03 | 0.19 | 1.1E-01 | 0.31 | 4.9E-02 | 0.14 | 5.6E-02 | 0.14 | 1.0E+00 | 0.00 |
| chr9 | 99413954 | rs144710877 | T/G | C9orf21 | p.101Y>S | 1/212 | 31/736 | 4.3E-03 | 0.11 | 2.8E-02 | 0.10 | 6.0E-02 | 0.15 | 7.6E-02 | 0.16 | 7.4E-02 | 0.15 |
| chr15 | 89453152 | rs143117049 | T/C | MFGE8 | p.26I>V | 1/212 | 31/736 | 4.3E-03 | 0.11 | 7.7E-02 | 0.16 | 6.1E-02 | 0.15 | 3.9E-02 | 0.12 | 2.2E-01 | 0.28 |
| chr2 | 54023133 | rs192316706 | A/G | ERLEC1 | p.111S>G | 1/214 | 30/736 | 7.1E-03 | 0.11 | 1.5E-01 | 0.23 | 6.5E-02 | 0.15 | 1.0E+00 | 0.00 | 1.0E+00 | 0.00 |
| chr8 | 25230071 | rs143521106 | C/G | DOCK5 | spliceSite | 1/212 | 30/736 | 7.0E-03 | 0.11 | 1.0E-01 | 0.18 | 6.5E-02 | 0.15 | 1.0E+00 | 0.00 | 1.0E+00 | 0.00 |
| chr1 | 155217643 | rs2072648 | C/T | FAM189B | p.646R>H | 1/212 | 30/736 | 7.0E-03 | 0.11 | 1.4E-02 | 0.08 | 6.7E-02 | 0.15 | 6.3E-02 | 0.15 | 3.6E-02 | 0.11 |
| chr19 | 36336398 | rs114615449 | C/G | NPHS1 | p.601G>A | 1/208 | 29/736 | 7.0E-03 | 0.12 | 3.0E-02 | 0.10 | 6.7E-02 | 0.15 | 4.1E-02 | 0.12 | 6.8E-02 | 0.14 |
| chr1 | 11008844 | rs117528334 | C/G | C1orf127 | p.283A>L | 1/214 | 30/736 | 7.1E-03 | 0.11 | 7.0E-02 | 0.15 | 7.4E-02 | 0.16 | 6.1E-02 | 0.15 | 2.2E-01 | 0.25 |
| chr8 | 19362969 | rs140161612 | G/A | CSGALNACT1 | p.126S>L | 11/212 | 10/736 | 2.3E-03 | 3.97 | 1.4E-02 | 3.23 | 1.5E-01 | 2.14 | 4.0E-03 | 3.90 | 2.0E-01 | 2.09 |
| chr7 | 44180676 | rs117394324 | G/A | MYL7 | spliceSite | 7/210 | 5/736 | 6.9E-03 | 5.04 | 5.5E-02 | 3.45 | 1.6E-01 | 2.84 | 5.8E-02 | 3.57 | 4.0E-01 | 1.93 |
| chr17 | 45786519 | rs369896558 | G/A | TBKBP1 | p.474A>T | 6/114 | 5/512 | 6.8E-03 | 5.63 | 3.8E-03 | 4.75 | 8.6E-01 | 1.18 | 2.5E-02 | 3.38 | 2.2E-01 | 3.78 |
| chr7 | 56128100 | rs192970041 | A/G | CCT6A | p.402I>V | 1/212 | 30/736 | 7.0E-03 | 0.11 | 9.7E-02 | 0.18 | 1.0E+00 | 0.00 | 4.6E-02 | 0.13 | 1.0E+00 | 0.00 |
| chr12 | 80169728 | . | A/C | PPP1R12A | p.938I>R | 15/210 | 0/160 | 2.2E-04 | 25.45 | NA | NA | NA | NA | NA | NA | NA | NA |
| chr9 | 32488848 | . | T/G | DDX58 | p.279E>D | 17/212 | 0/160 | 6.2E-05 | 28.73 | NA | NA | NA | NA | NA | NA | NA | NA |
| chr9 | 32480272 | . | A/T | DDX58 | p.573N>K | 15/212 | 0/160 | 2.2E-04 | 25.19 | NA | NA | NA | NA | NA | NA | NA | NA |
| chr9 | 32488815 | . | C/A | DDX58 | p.290K>N | 15/210 | 0/160 | 2.2E-04 | 25.45 | NA | NA | NA | NA | NA | NA | NA | NA |
| chr9 | 32480253 | . | C/T | DDX58 | p.580D>K | 14/212 | 0/160 | 4.3E-04 | 23.45 | NA | NA | NA | NA | NA | NA | NA | NA |
| chr9 | 32708645 | . | C/A | RP11-555J4.2 | spliceSite | 11/166 | 0/158 | 8.5E-04 | 23.44 | NA | NA | NA | NA | NA | NA | NA | NA |
| chr9 | 32467869 | . | A/C | DDX58 | p.692N>K | 13/212 | 0/160 | 8.2E-04 | 21.72 | NA | NA | NA | NA | NA | NA | NA | NA |
| chr14 | 102973415 | rs199786978 | G/C | ANKRD9 | p.271A>G | 11/188 | 0/160 | 1.2E-03 | 20.80 | NA | NA | NA | NA | NA | NA | NA | NA |
| chr9 | 32457310 | . | A/T | DDX58 | p.863F>Y | 11/212 | 0/160 | 3.1E-03 | 18.32 | NA | NA | NA | NA | NA | NA | NA | NA |
| chr9 | 32466410 | . | A/T | DDX58 | p.739F>I | 11/212 | 0/160 | 3.1E-03 | 18.32 | NA | NA | NA | NA | NA | NA | NA | NA |
| chr1 | 147092352 | rs200395772 | G/T | BCL9 | p.797L>F | 10/214 | 0/160 | 6.1E-03 | 16.48 | NA | NA | NA | NA | NA | NA | NA | NA |
| chr16 | 20359959 | . | G/T | UMOD | p.222R>S | 8/196 | 0/160 | 9.4E-03 | 14.47 | NA | NA | NA | NA | NA | NA | NA | NA |
| chr1 | 36298037 | . | G/A | EIF2C4 | spliceSite | 9/208 | 0/160 | 5.9E-03 | 15.29 | NA | NA | NA | NA | NA | NA | NA | NA |
| chr2 | 216237006 | rs76749241 | C/T | FN1 | p.1998V>I | 6/214 | 0/366 | 2.4E-03 | 22.85 | NA | NA | NA | NA | NA | NA | NA | NA |
| chr17 | 66890377 | rs530154281 | A/T | ABCA8 | p.951N>K | 6/212 | 0/366 | 2.3E-03 | 23.07 | NA | NA | NA | NA | NA | NA | NA | NA |
| chr5 | 134210196 | . | G/T | TXNDC15 | p.27G>* | 8/198 | 0/160 | 9.7E-03 | 14.32 | NA | NA | NA | NA | NA | NA | NA | NA |
| chr1 | 2560896 | . | T/C | MMEL1 | p.1M>V | 10/210 | 0/160 | 6.0E-03 | 16.81 | NA | NA | NA | NA | NA | NA | NA | NA |
| chr19 | 407689 | . | A/G | C2CD4C | p.225S>P | 9/194 | 0/148 | 6.1E-03 | 15.21 | NA | NA | NA | NA | NA | NA | NA | NA |
| chr17 | 65989048 | rs28368756 | T/C | C17orf58 | p.72E>G | 208/212 | 736/736 | 2.4E-03 | 0.03 | NA | NA | NA | NA | NA | NA | NA | NA |

Note: Summary statistics of the exome-wide variants were available at the AlzData webserver (http://www.alzdata.org/exome.html), which was established in our previous study .

Chr, chromosomal number

Position, chromosomal location of target variant according to hg19 (http://asia.ensembl.org/info/website/tutorials/grch37.html)

SNP_ID, rs# in dbSNP (https://www.ncbi.nlm.nih.gov/snp/)

Allele, reference allele / alterative allele

Gene, gene containing the target variant

Function, consequence of the target variant on protein coding

AC/AN_AD, allele count / total number of alleles in patients with Alzheimer’s disease

AC/AN_Ctrl, allele count / total number of alleles in healthy controls. The exome data of 160 in-house non-dementia individuals were pooled with the whole genome data of Han Chinese in Beijing (N=103) and Southern Han Chinese (N=105) from the 1000 Genome Project phase 3 as the initial population control (N = 368). The total number of alleles might be different for some variants, as the call rate for each variant varies due to different sequencing platform.

Fisher *P*, *P*-value of the Fisher’s exact test for allele frequency difference between cases and controls

Fisher OR, odds ratio of the Fisher’s exact test for the alternative allele relative to the reference allele

PC adj *P*, adjusted *P*-value based on the top three principal components as estimated in Supplementary Figure 1

PC adj OR, adjusted odds ratio for the alternative allele relative to the reference allele based on the top three principal components as estimated in Supplementary Figure 1

Sex adj *P*, adjusted *P*-value by sex

Sex adj OR, adjusted odds ratio for the alternative allele relative to the reference allele by sex

APOE adj *P*, adjusted *P*-value by APOE ε4 status

APOE adj OR, adjusted odds ratio for the alternative allele relative to the reference allele by APOE ε4 status

PC Sex APOE adj *P*, adjusted *P*-value with the top three principal components, sex, and APOE4 ε4 status as covariates

PC Sex APOE adj OR, adjusted odds ratio for the alternative allele relative to the reference allele, with the top three principal components, sex, and APOE4 ε4 status as covariates

**Supplementary Table 2. Association of *C7* variant rs3792646 with Alzheimer’s disease stratified by *APOE*** ε4 status

| **Sample ^1^** | **Allele** | **APOE ε4+** |  |  |  |  | **APOE ε4-** |  |  |  |  |
| --- | --- | --- | --- | --- | --- | --- | --- | --- | --- | --- | --- |
|  |  | **Alzheimer** | **Control** | ***P*-value^2^** | **OR** | **95% CI** | **Alzheimer** | **Control** | ***P*-value^2^** | **OR** | **95% CI** |
| **WES** | C | 10 | 1 | **0.00072** | 16.143 | 2.022-128.850 | 6 | 7 | **0.01385** | 4.286 | 1.416-12.972 |
|  | A | 70 | 113 |  |  |  | 123 | 615 |  |  |  |
| **North-Beijing** | C | 4 | 1 | **0.03052** | 9.826 | 1.069-90.291 | 3 | 7 | **0.04973** | 4.624 | 1.164-18.371 |
|  | A | 46 | 113 |  |  |  | 57 | 615 |  |  |  |
| **East** | C | 16 | 3 | 0.77767 | 1.34 | 0.38281-4.692 | 21 | 4 | **0.00375** | 4.291 | 1.462-12.595 |
|  | A | 386 | 97 |  |  |  | 531 | 434 |  |  |  |
| **Southwest** | C | 10 | 9 | **0.01475** | 3.214 | 1.291-8.000 | 17 | 76 | 0.11594 | 1.54 | 0.90061-2.632 |
|  | A | 252 | 729 |  |  |  | 401 | 2760 |  |  |  |
| **Southcentral** | C | 6 | 9 | **0.03739** | 3.115 | 1.093-8.879 | 10 | 76 | 0.579 | 1.219 | 0.62355-2.382 |
|  | A | 156 | 729 |  |  |  | 298 | 2760 |  |  |  |
| **Combined** | C | 46 | 13 | **1.435E-05** | 3.651 | 1.960-6.803 | 57 | 87 | **0.00122** | 1.77 | 1.260-2.485 |
|  | A | 910 | 939 |  |  |  | 1410 | 3809 |  |  |  |

Note: A allele, reference allele; C allele, risk allele; *P*-value, Fisher’s exact test for allele frequency difference between cases and controls; OR, Odds ratio of the Fisher’s exact test for the alternative allele relative to the reference allele; 95% CI, 95% confidence interval

**^1^** Only individuals with genotyping information for both rs3792646 and the APOE ε4 status were included in the analyses

**^2^** *P-values* < 0.05 were marked in bold.

**Supplementary Table 3. No association of *C7* variants with Alzheimer’s disease in the ADNI European individuals**

| **Chr: Position** | **Allele** | **SNP** | **F_A** | **F_U** | ***P*** | **OR** | **Annotation** | **Mutation** | **Prediction** |
| --- | --- | --- | --- | --- | --- | --- | --- | --- | --- |
| 5:40909694 | T/C | kgp22074974 | 0.000 | 0.004 | 0.146 | 0.000 | 5’utr | - | - |
| 5:40936541 | C/T | kgp22327897 | 0.007 | 0.002 | 0.198 | 3.816 | Coding | p.C128R | Probably damaging |
| 5:40945397 | A/G | kgp22664496 | 0.002 | 0.004 | 0.533 | 0.474 | Coding | p.R222H | Possibly damaging |
| 5:40955561 | C/G | rs1063499 | 0.394 | 0.427 | 0.248 | 0.871 | Coding | p.S389T | Probably damaging |
| 5:40955653 | C/A | rs3792646 | 0.002 | 0.000 | 0.330 | NA | Coding | p.K420Q | Damaging |
| 5:40964852 | C/A | rs13157656 | 0.233 | 0.230 | 0.886 | 1.020 | Coding | p.T587P | Benign |
| 5:40981927 | G/A | kgp3954039 | 0.019 | 0.027 | 0.354 | 0.690 | 3’utr | - | - |
| 5:40982031 | C/T | rs10473230 | 0.159 | 0.155 | 0.853 | 1.031 | 3’utr | - | - |
| 5:40982175 | C/T | kgp11519444 | 0.213 | 0.226 | 0.590 | 0.926 | 3’utr | - | - |
| 5:40982620 | T/G | kgp6970181 | 0.210 | 0.210 | 0.983 | 0.997 | 3’utr | - | - |
| 5:40982622 | T/G | rs1061443 | 0.208 | 0.210 | 0.927 | 0.987 | 3’utr | - | - |
| 5:40982780 | G/A | rs8264 | 0.159 | 0.155 | 0.853 | 1.031 | 3’utr | - | - |
| 5:40982977 | G/A | kgp9618283 | 0.208 | 0.210 | 0.927 | 0.987 | 3’utr | - | - |

Note: Data were retrieved from the ADNI WGS phase (http://adni.loni.usc.edu/) . Allele, reference allele/alternative allele; SNP: rs number in dbSNP dataset and the original SNP number (labeled with “kgp”) in the ADNI dataset; F_A, allele frequency in patients (N = 296); F_U, allele frequency in controls (N = 281); *P*, Fisher’s exact test *P*-value, OR, odds ratio; utr, untranslated region. Data were processed by using PLINK . Variants annotation was performed by the web tool SNPnexus (http://snp-nexus.org/index.html). For missense substitution, we provided the predicted effect on protein function (possibly damaging, probably damaging, and benign) based on the PolyPhen program .

**Supplementary Table 4. mRNA expression pattern of the complement components in frontal cortex tissues of patients with Alzheimer’s disease compared with controls**

| **Catalog** | **Gene** | ***P-value*** | **Log_2_FC** |
| --- | --- | --- | --- |
| **Initial** | ***C1QA*** | **1.80×10^-18^** | 0.313052 |
|  | ***C1QB*** | **1.07×10^-15^** | 0.236765 |
|  | ***C1QC*** | **1.90×10^-13^** | 0.258328 |
|  |  |  |  |
| **Central** | ***C2*** | **8.45×10^-04^** | 0.068271 |
|  | ***C3*** | **4.01×10^-09^** | 0.19697 |
|  |  |  |  |
| **Terminal** | *C5* | 8.26×10^-1^ | -0.00303 |
|  | *C6* | 5.44×10^-1^ | 0.011006 |
|  | ***C7*** | **3.21×10^-15^** | 0.242259 |
|  | *C8B* | 4.11×10^-1^ | 0.017471 |
|  | *C8G* | 4.37×10^-2^ | 0.017017 |
|  |  |  |  |
| **Regulator** | ***C1QBP*** | **1.95×10^-10^** | -0.06962 |
|  | *CR2* | 3.29×10^-2^ | 0.018832 |
|  | ***C3AR1*** | **6.39×10^-17^** | 0.250126 |
|  | *C4BPA* | 8.83×10^-2^ | 0.069149 |
|  | *C4BPB* | 8.33×10^-1^ | 0.003635 |
|  | *CFHR3* | 8.81×10^-3^ | -0.0173 |
|  | *CFHR4* | 9.32×10^-1^ | 0.000676 |
|  | ***CFHR5*** | **7.18×10^-5^** | -0.05121 |
|  | ***CFD*** | **4.10×10^-6^** | 0.063789 |
|  | ***CFP*** | **2.51×10^-3^** | 0.030998 |

Note: Data were retrieved from GSE33000, which is the largest individual data set of frontal cortex tissues from patients with Alzheimer’s disease and controls . Differential expression *P*-values were calculated by using the *limma* package of R. Log_2_FC, log of fold change for target genes in patients compared with controls. Full profiles are available at our newly established webserver AlzData ([www.alzdata.org](http://www.alzdata.org/)) . *P-values* less than the threshold of Bonferroni correction for 20 genes (*P*_corrected_ = 2.50×10^-3^) are marked in bold.

**Acknowledgment statement for the ADSP**

The Alzheimer’s Disease Sequencing Project (ADSP) is comprised of two Alzheimer’s Disease (AD) genetics consortia and three National Human Genome Research Institute (NHGRI) funded Large Scale Sequencing and Analysis Centers (LSAC). The two AD genetics consortia are the Alzheimer’s Disease Genetics Consortium (ADGC) funded by NIA (U01 AG032984), and the Cohorts for Heart and Aging Research in Genomic Epidemiology (CHARGE) funded by NIA (R01 AG033193), the National Heart, Lung, and Blood Institute (NHLBI), other National Institute of Health (NIH) institutes and other foreign governmental and non-governmental organizations. The Discovery Phase analysis of sequence data is supported through UF1AG047133 (to Drs. Schellenberg, Farrer, Pericak-Vance, Mayeux, and Haines); U01AG049505 to Dr. Seshadri; U01AG049506 to Dr. Boerwinkle; U01AG049507 to Dr. Wijsman; and U01AG049508 to Dr. Goate and the Discovery Extension Phase analysis is supported through U01AG052411 to Dr. Goate, U01AG052410 to Dr. Pericak-Vance and U01 AG052409 to Drs. Seshadri and Fornage. Data generation and harmonization in the Follow-up Phases is supported by U54AG052427 (to Drs. Schellenberg and Wang).

The ADGC cohorts include: Adult Changes in Thought (ACT), the Alzheimer’s Disease Centers (ADC), the Chicago Health and Aging Project (CHAP), the Memory and Aging Project (MAP), Mayo Clinic (MAYO), Mayo Parkinson’s Disease controls, University of Miami, the Multi-Institutional Research in Alzheimer’s Genetic Epidemiology Study (MIRAGE), the National Cell Repository for Alzheimer’s Disease (NCRAD), the National Institute on Aging Late Onset Alzheimer's Disease Family Study (NIA-LOAD), the Religious Orders Study (ROS), the Texas Alzheimer’s Research and Care Consortium (TARC), Vanderbilt University/Case Western Reserve University (VAN/CWRU), the Washington Heights-Inwood Columbia Aging Project (WHICAP) and the Washington University Sequencing Project (WUSP), the Columbia University Hispanic- Estudio Familiar de Influencia Genetica de Alzheimer (EFIGA), the University of Toronto (UT), and Genetic Differences (GD).

The CHARGE cohorts are supported in part by National Heart, Lung, and Blood Institute (NHLBI) infrastructure grant HL105756 (Psaty), RC2HL102419 (Boerwinkle) and the neurology working group is supported by the National Institute on Aging (NIA) R01 grant AG033193. The CHARGE cohorts participating in the ADSP include the following: Austrian Stroke Prevention Study (ASPS), ASPS-Family study, and the Prospective Dementia Registry-Austria (ASPS/PRODEM-Aus), the Atherosclerosis Risk in Communities (ARIC) Study, the Cardiovascular Health Study (CHS), the Erasmus Rucphen Family Study (ERF), the Framingham Heart Study (FHS), and the Rotterdam Study (RS). ASPS is funded by the Austrian Science Fond (FWF) grant number P20545-P05 and P13180 and the Medical University of Graz. The ASPS-Fam is funded by the Austrian Science Fund (FWF) project I904),the EU Joint Programme - Neurodegenerative Disease Research (JPND) in frame of the BRIDGET project (Austria, Ministry of Science) and the Medical University of Graz and the Steiermärkische Krankenanstalten Gesellschaft. PRODEM-Austria is supported by the Austrian Research Promotion agency (FFG) (Project No. 827462) and by the Austrian National Bank (Anniversary Fund, project 15435. ARIC research is carried out as a collaborative study supported by NHLBI contracts (HHSN268201100005C, HHSN268201100006C, HHSN268201100007C, HHSN268201100008C, HHSN268201100009C, HHSN268201100010C, HHSN268201100011C, and HHSN268201100012C). Neurocognitive data in ARIC is collected by U01 2U01HL096812, 2U01HL096814, 2U01HL096899, 2U01HL096902, 2U01HL096917 from the NIH (NHLBI, NINDS, NIA and NIDCD), and with previous brain MRI examinations funded by R01-HL70825 from the NHLBI. CHS research was supported by contracts HHSN268201200036C, HHSN268200800007C, N01HC55222, N01HC85079, N01HC85080, N01HC85081, N01HC85082, N01HC85083, N01HC85086, and grants U01HL080295 and U01HL130114 from the NHLBI with additional contribution from the National Institute of Neurological Disorders and Stroke (NINDS). Additional support was provided by R01AG023629, R01AG15928, and R01AG20098 from the NIA. FHS research is supported by NHLBI contracts N01-HC-25195 and HHSN268201500001I. This study was also supported by additional grants from the NIA (R01s AG054076, AG049607 and AG033040 and NINDS (R01 NS017950). The ERF study as a part of EUROSPAN (European Special Populations Research Network) was supported by European Commission FP6 STRP grant number 018947 (LSHG-CT-2006-01947) and also received funding from the European Community's Seventh Framework Programme (FP7/2007-2013)/grant agreement HEALTH-F4-2007-201413 by the European Commission under the programme "Quality of Life and Management of the Living Resources" of 5th Framework Programme (no. QLG2-CT-2002-01254). High-throughput analysis of the ERF data was supported by a joint grant from the Netherlands Organization for Scientific Research and the Russian Foundation for Basic Research (NWO-RFBR 047.017.043). The Rotterdam Study is funded by Erasmus Medical Center and Erasmus University, Rotterdam, the Netherlands Organization for Health Research and Development (ZonMw), the Research Institute for Diseases in the Elderly (RIDE), the Ministry of Education, Culture and Science, the Ministry for Health, Welfare and Sports, the European Commission (DG XII), and the municipality of Rotterdam. Genetic data sets are also supported by the Netherlands Organization of Scientific Research NWO Investments (175.010.2005.011, 911-03-012), the Genetic Laboratory of the Department of Internal Medicine, Erasmus MC, the Research Institute for Diseases in the Elderly (014-93-015; RIDE2), and the Netherlands Genomics Initiative (NGI)/Netherlands Organization for Scientific Research (NWO) Netherlands Consortium for Healthy Aging (NCHA), project 050-060-810. All studies are grateful to their participants, faculty and staff. The content of these manuscripts is solely the responsibility of the authors and does not necessarily represent the official views of the National Institutes of Health or the U.S. Department of Health and Human Services.

The four LSACs are: the Human Genome Sequencing Center at the Baylor College of Medicine (U54 HG003273), the Broad Institute Genome Center (U54HG003067), The American Genome Center at the Uniformed Services University of the Health Sciences (U01AG057659), and the Washington University Genome Institute (U54HG003079).

Biological samples and associated phenotypic data used in primary data analyses were stored at Study Investigators institutions, and at the National Cell Repository for Alzheimer’s Disease (NCRAD, U24AG021886) at Indiana University funded by NIA. Associated Phenotypic Data used in primary and secondary data analyses were provided by Study Investigators, the NIA funded Alzheimer’s Disease Centers (ADCs), and the National Alzheimer’s Coordinating Center (NACC, U01AG016976) and the National Institute on Aging Genetics of Alzheimer’s Disease Data Storage Site (NIAGADS, U24AG041689) at the University of Pennsylvania, funded by NIA, and at the Database for Genotypes and Phenotypes (dbGaP) funded by NIH. This research was supported in part by the Intramural Research Program of the National Institutes of health, National Library of Medicine. Contributors to the Genetic Analysis Data included Study Investigators on projects that were individually funded by NIA, and other NIH institutes, and by private U.S. organizations, or foreign governmental or nongovernmental organizations.

**References**

1. 1000 Genomes Project Consortium, Auton A, Brooks LD, et al.; A global reference for human genetic variation. *Nature* 2015; **526**(7571):68-74.

2. Wang D, Fan Y, Malhi M, et al.; Missense variants in HIF1A and LACC1 contribute to leprosy risk in Han Chinese. *Am J Hum Genet* 2018; **102**:794-805.

3. Weiner MW, Aisen PS, Jack CR, Jr., et al.; The Alzheimer's disease neuroimaging initiative: progress report and future plans. *Alzheimers Dement* 2010; **6**(3):202-211.e7.

4. Purcell S, Neale B, Todd-Brown K, et al.; PLINK: a tool set for whole-genome association and population-based linkage analyses. *Am J Hum Genet* 2007; **81**(3):559-75.

5. Matarin M, Salih DA, Yasvoina M, et al.; A genome-wide gene-expression analysis and database in transgenic mice during development of amyloid or tau pathology. *Cell Rep* 2015; **10**(4):633-44.

6. Xu M, Zhang DF, Luo R, et al.; A systematic integrated analysis of brain expression profiles reveals YAP1 and other prioritized hub genes as important upstream regulators in Alzheimer's disease. *Alzheimers Dement* 2018; **14**:215-229.

7. Adzhubei IA, Schmidt S, Peshkin L, et al.; A method and server for predicting damaging missense mutations. *Nat Methods* 2010; **7**(4):248-9.

8. Narayanan M, Huynh JL, Wang K, et al.; Common dysregulation network in the human prefrontal cortex underlies two neurodegenerative diseases. *Mol Syst Biol* 2014; **10**:743.
